# Electrically conductive carbon‐based (bio)‐nanomaterials for cardiac tissue engineering

**DOI:** 10.1002/btm2.10347

**Published:** 2022-06-21

**Authors:** Negin Jalilinejad, Mohammad Rabiee, Nafiseh Baheiraei, Ramin Ghahremanzadeh, Reza Salarian, Navid Rabiee, Omid Akhavan, Payam Zarrintaj, Aleksander Hejna, Mohammad Reza Saeb, Ali Zarrabi, Esmaeel Sharifi, Satar Yousefiasl, Ehsan Nazarzadeh Zare

**Affiliations:** ^1^ Biomaterial Group, Department of Biomedical Engineering Amirkabir University of Technology Tehran Iran; ^2^ Tissue Engineering and Applied Cell Sciences Division, Department of Anatomical Sciences, Faculty of Medical Sciences Tarbiat Modares University Tehran Iran; ^3^ Nanobiotechnology Research Center Avicenna Research Institute, ACECR Tehran Iran; ^4^ Biomedical Engineering Department Maziar University Royan Mazandaran Iran; ^5^ Department of Physics Sharif University of Technology Tehran Iran; ^6^ School of Engineering Macquarie University Sydney New South Wales Australia; ^7^ Department of Materials Science and Engineering Pohang University of Science and Technology (POSTECH), 77 Cheongam‐ro, Nam‐gu Pohang Gyeongbuk South Korea; ^8^ School of Chemical Engineering Oklahoma State University Stillwater Oklahoma USA; ^9^ Department of Polymer Technology, Faculty of Chemistry Gdańsk University of Technology Gdańsk Poland; ^10^ Department of Biomedical Engineering, Faculty of Engineering and Natural Sciences Istinye University Istanbul Turkey; ^11^ Department of Tissue Engineering and Biomaterials, School of Advanced Medical Sciences and Technologies Hamadan University of Medical Sciences Hamadan Iran; ^12^ School of Dentistry Hamadan University of Medical Sciences Hamadan Iran; ^13^ School of Chemistry Damghan University Damghan Iran

**Keywords:** carbon‐based biomaterials, cardiac tissue engineering, graphene, graphene oxide, scaffolds, stem cells

## Abstract

A proper self‐regenerating capability is lacking in human cardiac tissue which along with the alarming rate of deaths associated with cardiovascular disorders makes tissue engineering critical. Novel approaches are now being investigated in order to speedily overcome the challenges in this path. Tissue engineering has been revolutionized by the advent of nanomaterials, and later by the application of carbon‐based nanomaterials because of their exceptional variable functionality, conductivity, and mechanical properties. Electrically conductive biomaterials used as cell bearers provide the tissue with an appropriate microenvironment for the specific seeded cells as substrates for the sake of protecting cells in biological media against attacking mechanisms. Nevertheless, their advantages and shortcoming in view of cellular behavior, toxicity, and targeted delivery depend on the tissue in which they are implanted or being used as a scaffold. This review seeks to address, summarize, classify, conceptualize, and discuss the use of carbon‐based nanoparticles in cardiac tissue engineering emphasizing their conductivity. We considered electrical conductivity as a key affecting the regeneration of cells. Correspondingly, we reviewed conductive polymers used in tissue engineering and specifically in cardiac repair as key biomaterials with high efficiency. We comprehensively classified and discussed the advantages of using conductive biomaterials in cardiac tissue engineering. An overall review of the open literature on electroactive substrates including carbon‐based biomaterials over the last decade was provided, tabulated, and thoroughly discussed. The most commonly used conductive substrates comprising graphene, graphene oxide, carbon nanotubes, and carbon nanofibers in cardiac repair were studied.

## 1 INTRODUCTION

Drug treatments are efficient mainly in the case of limited minor injuries, while most extensive and progressive damages to tissues and subsequent loss of organ functions are much more severe.^1^, ^2^ Such conditions bring about the possibility of organ failure, which may require a completely functional replacement. In other words, progressive tissue loss for various reasons and the high necessity of consistent proper organ function bring about an urgent need for a complete replacement. Almost inadequate regenerative capability of the human body brings about significant consideration over other attitudes. Despite all the limitations and risks, organ transplant seems to be significantly efficient. However, an average of 20 people die every day while waiting for an organ transplant, as reported by the U.S. Department of Health and Human Services.^3^, ^4^, ^5^, ^6^, ^7^

Other than organ transplant and pharmaceutical approaches, surgical reconstruction procedures effectively aim for tissue repair. It mainly focuses on controlling inflammation,^8^, ^9^, ^10^ reducing scar formation,^11^, ^12^ and identifying cures for fibrotic diseases and chronic wounds.^13^, ^14^ Total artificial substitutes (such as artificial joints) and nonliving processed tissues (such as heart valves) are the replacing strategies that are pleasingly efficient. In addition, harvested flaps (including autografts or allografts) are conventional strategies associated with reconstruction perspectives.^15^, ^16^ However, harvesting autografts is usually accompanied by challenges of formidable donor site morbidity. Besides, it requires multiple separate operations, which is preferably avoided. On the other hand, in the course of applying grafts, precisely transplanting vasculatures to the target site demands highly accurate and advanced equipment. High risks of infection or disease in case of allotransplantation can also arise.^17^ Hence, surgical reconstruction procedures as well, may not be the method of choice when it comes to urgent and critical situations.

Tissue engineering is a promising technique aiming at tissue reconstruction through regeneration. The three main approaches are cell transplantation, matrix‐guided regeneration, and simultaneous utilization of both cells within matrices. Nowadays, tissue engineering employs an optimal combination of cells, substrates, and bioactive molecules to alleviate lost tissues.^15^, ^18^, ^19^

Scaffolds are assumed to act as a matrix aiming to satisfy several demands, primarily providing the initial cell support.^20^ They are supposed to adhere to cells via ligands and chemical groups/compounds of atoms.^21^, ^22^, ^23^ Thus, hydrophilic materials,^24^, ^25^ porous structures,^26^, ^27^, ^28^ and large specific surface areas^29^ efficiently facilitate cell adhesion. New blood vessel formation, including vasculogenesis and angiogenesis, is also a critical challenge that promotes cell survival and enables the operation of larger tissues. Previously mentioned demands and pores interconnectivity, which promote mass transport including oxygen and nutrient transfer, and the integration of the implant to the adjacent area are likely to be associated with interactions with the microenvironment.^30^, ^31^, ^32^ Moreover, scaffolds and matrices are supposed to function as mechanical support during tissue formation, whether in vitro or in vivo. Therefore, an appropriate elasticity and stability in the case of either soft or hard tissue are essential. Optimization between the density, porosity, and mechanical properties of the scaffold is of great importance as a consequence. Apart from this, while tissue growth and adhesion occur, concurrent degradation of the scaffold occurs with extracellular matrix proteins replacing it.^33^ Nontoxicity and ease of absorbance or excretion of degradation products are other vital factors.

Applied materials acting as cell bearers are extremely fundamental because of different reasons. As indicated, they are employed as supporting substrates acting as an appropriate microenvironment for the specific seeded cells. Additionally, a substrate protects cells from being recognized by the immune system^34^ and neutrophils attack^34^, ^35^ of the patient's body. Depending on the native tissue, the composition, elasticity, and microstructure of the extracellular matrix (ECM) differ explicitly from tissue to tissue and even in different periods of one specific tissue. Consequently, it has been demonstrated that cultured cells on various substrates with differing features show various responses.^36^ Thus, to eventually accomplish the expected cellular behavior, multiple parameters should be regarded.^20^

Considering every stated parameter, deciding on an appropriate substrate in tissue engineering is of tremendous importance. Acellular tissue matrices, biocompatible natural or synthetic polymers, ceramics and their composition, and recently graphene‐based materials are considered suitable choices for the substrates. Material selection is based on the application, cell‐scaffold interactions, appropriate mechanical and electrical properties, required time of the scaffold performance before degradation, and the feasible fabrication methods.^37^, ^38^, ^39^, ^40^, ^41^

This review aims to provide a survey on cardiac tissue engineering and the significance of conductivity at the same time. First, electrical conductivity was defined and different aspects of such characteristics in nanomedicine were discussed. Then, conductive polymers used in tissue engineering, particularly in cardiac repair, were comprehensively classified and their advantages in cardiac tissue engineering were highlighted. As the main objective of this work, an overall review of studies on electroactive substrates comprising carbon‐based materials within the past few years was reported, tabulated, and discussed. In this regard, the most frequently used carbon‐based substrates including graphene, graphene oxide, carbon nanotubes, and carbon nanofibers in cardiac repair were studied.

## 2 CARDIAC TISSUE REGENERATION

Different biomaterials and their combinations are currently under investigation for tissue engineering.^42^ Decellularized tissues have been widely used as either cell seeding or cell‐free substrates.^27^ Extracellular matrix‐derived materials are beneficial since they provide a native microenvironment for the specific cells to survive, proliferate, and differentiate.^43^ Native ECM mixture supplies specific molecules and proper structure, promoting cell phenotype and maintaining tissue‐specific ECM construction. However, ECM variations originating from differing donors, immunologic and inflammatory response of the recipient, possible rejection of the implant, and regulatory issues, are the topics of limitation.^19^

Naturally‐occurring polymers, on the other hand, can be extracted from living organisms. Collagen, cellulose, alginate, silk fibroin, and chitosan^44^ are among the favored natural polymers typically used in this field. These polymers are beneficial due to their biological inherent mimicking of natural ECM structure.^45^ In return, lack of proper mechanical strength and hardly controllable degradation rate is a considerable drawback of natural materials. Apart from that, potential contaminants may be presented within the structure of natural polymers (S. J. Lee et al., 2018), such as heavy metals, formaldehyde, polyphenolic compounds, and bacteria, and this brings about the possibility of pathogenic behavior for such an eventually.^46^

Natural polymers and acellular matrices are beneficial in the aspect of biological recognition; while synthetic biomaterials provide the potential for more flexible and controllable properties such as mechanical characteristics and degradation rate.^47^ Mostly applied synthetic polymers used for tissue engineering include poly(ethylene glycol) (PEG), poly(ε‐caprolactone) (PCL), poly(lactide) acid (PLA), poly(lactic‐co‐glycolic acid) (PLGA), and polyurethane (PU). They are advantageous in the aspect of flexible physical and chemical properties. However, potential cytotoxicity due to lack of biological inherency is a disadvantage likely to promote cell reaction.^48^

Due to their substantial potential for osteoconductivity, ceramics are the material of choice for repairing and regenerating musculoskeletal and periodontal disorders. This is due to ceramics' fine biological and mechanical properties such as biocompatibility, hardness, and corrosion resistivity.^49^ Major hurdles in employing ceramics as substrates are attributed to their brittleness and high Young's modulus, making them difficult to process.^50^ Bioceramics are generally serving as in three categories. Bioinert ceramics such as alumina and zirconia are used when no interaction between the implant and the environs is preferred and is ascribed to the relatively high corrosion and wear resistance. Bioactive ceramics, on the other hand, gradually join their surroundings through osteogenesis. Bioactive glasses and glass ceramics are grouped into this category. Lastly, biodegradable ceramics, resorbed within the body over time, such as calcium phosphate‐based ceramics, are known as bioresorbable ceramics.^51^

Cardiovascular diseases (CVDs) are undoubtedly the primary cause of death globally, in recent years.^52^ Nearly 18 million deaths in 2016 were attributed to CVDs which is around 31% of all deaths. Except for the significant health threat, CVDs are a significant economic burden. According to the statistics, CVDs consume 14% of the USA health care cost annually, so 189.7 billion $ has been directly spent from 2012 to 2013 on the CVDs for direct expenditure with respect to 316.1 billion $ spent indirectly. It is anticipated that an unbelievable budget of about 918 billion $ will be demanded for the CVDs by 2030.^53^

Many CVDs are identified nowadays, such as stroke, rhythm disorders, heart failure, cognitional heart disease, and atherosclerosis. Congenital heart defects are among the most common congenital disabilities.^54^ More than 24% of infants dying due to a congenital disability suffer from congenital cardiovascular defects. Aside from that, coronary artery disease (CAD), also known as ischemic heart disease, is the most common class of CVDs^55^ associated with a partial blockage in major coronary arteries due to atherosclerosis.

In early diagnosis, coronary artery disease is finely treated with the percutaneous coronary intervention technique, also known as angioplasty. It is a nonsurgical procedure in which a catheter is inserted into blood vessels (usually the femoral artery in the thigh) and guided up toward the heart into the considered coronary artery. A balloon catheter is then pushed into the area and inflated to pull over the blockage and widen the vessel. Finally, a stent is placed to ensure the vessel remains extended since the balloon is ejected.^56^

Coronary artery bypass grafts may be required if the situation is more severe. An occluded coronary artery is bypassed utilizing an isolated artery or vein graft through a surgical procedure. The graft, usually harvested from the patient's leg or chest, is transplanted into the area with inadequate blood supply to provide a new pathway for blood flow. The heart may need to stop beating during the procedure and be timely replaced by a heart‐lung machine.^56^

As the blockage gradually intensifies, a severe occlusion typically forms if not diagnosed or treated properly, causing a significant heart attack due to improper expansion and contraction of the myocardium. After a heart attack, fibrotic scar tissue will be generated because of the limited capacity of myocardial tissue in inherent regeneration, which such incapability ends in left ventricular dysfunction and cardiac arrhythmias.^57^

Myocardial infarction (MI) occurs due to progressive, disrupted coronary circulation and unstable angina—deficiency of blood flow within the heart muscle. It is followed by a significant loss of cells in the dedicated area in response to oxygen demand and supply imbalance.^58^ The amount of involved area depends on the size of the coronary artery, the occlusion severity and duration, and the level of demanded oxygen by the involved myocardium.

Local cell death occurs due to the inadequacy of blood supply and oxygen shortage, ischemia in shorts. This occurs through the entire or part of the myocardium thickness within the involved area. The body's inflammatory response immediately begins,^13^ aiming for tissue repair leading to prompt healing. Cardiac muscle is explicitly made up of different cell types assorted as myocytes and non‐myocytes, including cardiomyocytes (CMs), fibroblasts, endothelial cells, and peri‐vascular cells. Although 70%–85% of the volume of cardiac muscle is occupied by CMs, these cells represent only 30% of the whole cardiac cell population.^59^ Hence, the inability of cardiac muscles to self‐regenerate is likely attributed to the CMs limited capability to proliferate in practice.^60^ In other words, an abundant potential for renewal lacks within cardiac contractile cells.^61^ Since the constant function of the myocardium is crucial,^41^, ^52^ healing rapidly recovers the deficiency of lost cells to compensate for the insufficiency. Thus, necrotic tissue formation and collagen deposition overtake regeneration, taking part in the healing process.^62^

**FIGURE 1:**
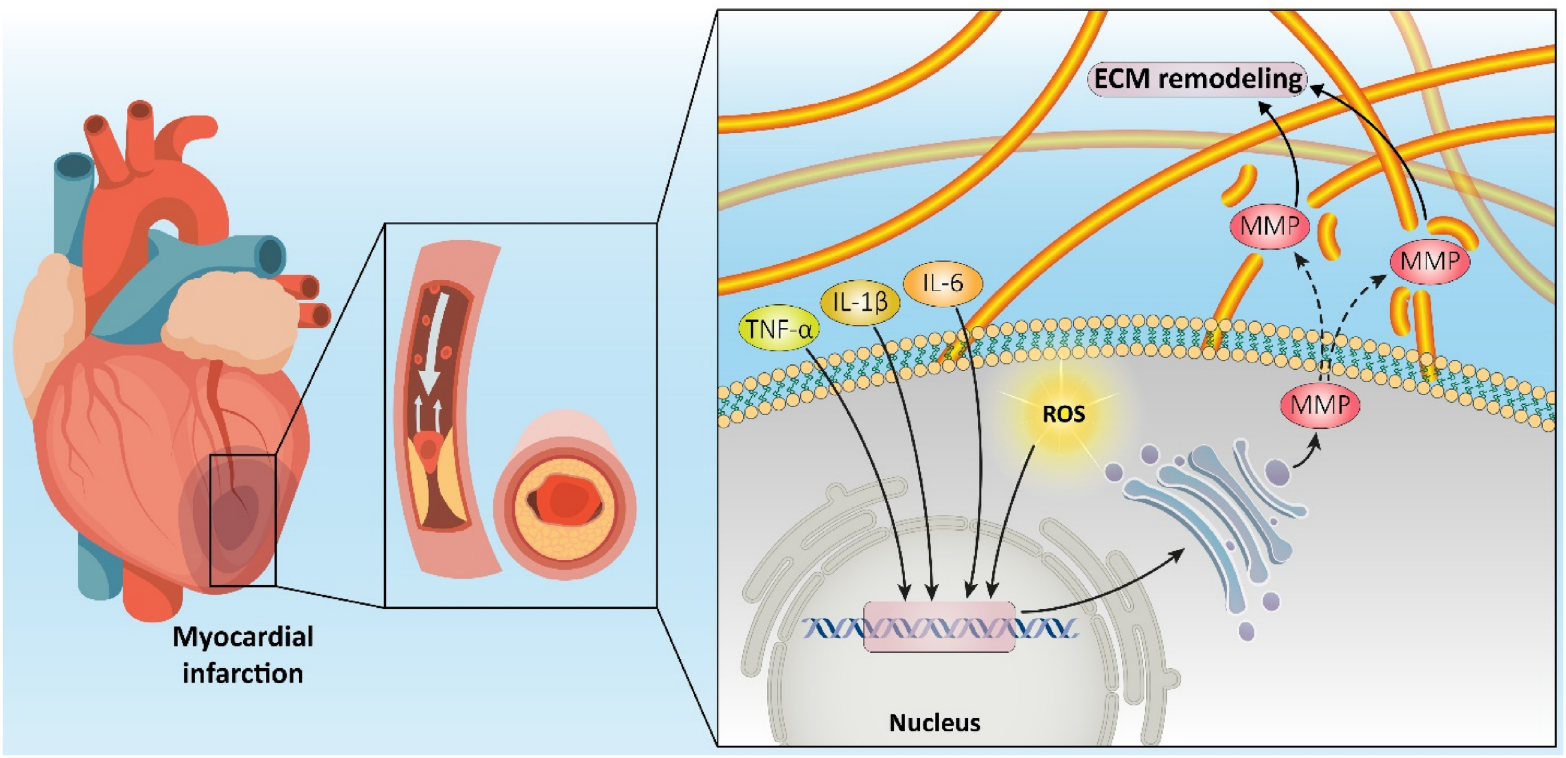
The molecular events occurring during myocardial infarction include ROS overgeneration that can lead to oxidative stress, and enhanced levels of cytokines such as TNF‐α, IL‐6, and IL‐1β for mediating inflammation. Furthermore, MMPs can mediate ECM remodeling during myocardial infarction.

Shortly after MI, several inflammatory responses are followed. In brief, oxidative stress, represented as enhanced generation of oxygen radicals or reactive oxygen species (ROS), is rapidly established. Meanwhile, inflammatory cytokines such as TNF‐α, IL‐1β, and IL‐6 are produced (Figure 1). Subsequently, cardio depressive reactions take place. Furthermore, the activation of matrix metalloproteinase (MMP) enables ECM remodeling. After that, collagenous tissue formation and fibrosis take place. Myocardium remodeling and LV dilation are also long‐term outcomes.^63^

As a result of cell necrosis, the body's inflammatory response is accompanied by ECM degradation to elucidate the indicated phenomena. This allows neutrophils and macrophages migration to the infarcted area. Phagocytosis of necrotic cells takes place as a consequence. Later, proliferating fibroblasts and endothelial cells establishing the granulation tissue replace necrotic cells.^64^, ^65^ Healing continues with the transformation of granulated tissue into scar tissue within a month or so. Rearrangement of cells and ECM to compensate for the injury causes a disturbance in the integrated electrophysiological performance of cardiac muscle. Given the aforementioned subsequent circumstances, uncoupled, dense, collagen‐rich scar tissue with independent mechanical and electrical properties than natural myocardium appears. Changes in ion channels and intercellular gap junctions are followed. Accordingly, lost integrity through the electrical activity of heart muscle causes a cardiac rhythm disturbance.^13^, ^35^

Taken together, these impairments lead to the insufficient capacity of the heart muscle to pump enough blood throughout the whole body. Due to the previously mentioned circumstances, mechanical stress brings about several permanent outcomes, including ventricle enlargement, heart wall thinning, geometry change, and LV dilation. LV chamber gradually encounters a minor conversion in its overall shape from ellipsoidal to spherical. This possibly leads to mitral regurgitation. Changes in the cavity diameter, mass, and geometry of the heart muscle bring about adverse impacts and deficiencies in cardiac performance. If so, chronic heart failure is likely to be inevitable then.^63^, ^66^

Drug treatments for patients either already suffering from or likely to face such difficulties within the near future, including beta‐blockers, ACE inhibitors, and angiotensin receptor blockers, are the most common treatments. However, they are not efficient enough, counted as inhibitors of LV dilation.^63^, ^67^, ^68^, ^69^, ^70^ Total heart transplantation, on the other hand, is by far a satisfying approach though insufficient donors, heightened risks of open‐heart procedure, probability of organ rejection, complex postoperative cares, and precautions of immunosuppression regimens are still considerable challenges making this approach extremely complicated.^34^, ^71^, ^72^, ^73^, ^74^ Moreover, such surgical operations carry significant risk to older people, which suffer from CVDs more often than younger patients do. Therefore, alternative regeneration routes have emerged to repair heart function appropriately. New emerging methods should be noninvasive (eliminating heart surgery), affordable, efficient, and appropriate for mimicking cardiac tissue.

**FIGURE 2:**
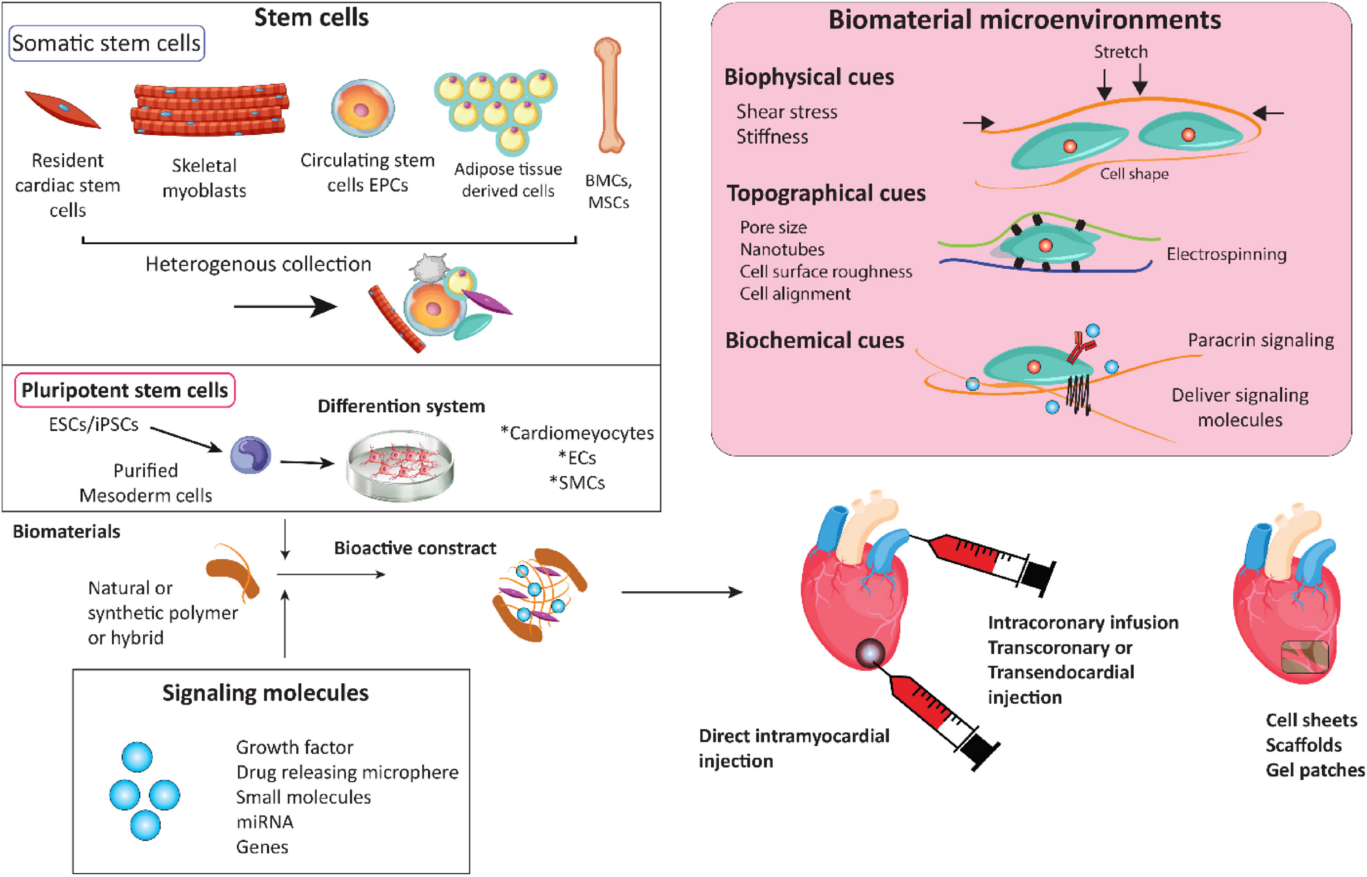
Cardiac tissue engineering strategies. Cells, scaffolds, and signaling molecules can be introduced alone or in combination at the injury site. Scaffolds provide biophysical, topographical, and biochemical microenvironments to the transplanted and host cells. The mechanical stiffness of biomaterials can guide proper stem cell differentiation. Stretch is a typical function of the cardiovascular system and has been shown to guide the differentiation of stem cells toward cardiomyocytes (CMs) or smooth muscle cells. Nanotopography of the biomaterial can affect stem cell phenotype, cellular alignment, and electrophysical properties^81^

As a solution to this unmet demand, myocardial tissue engineering with the aim of cardiac regeneration raises the chance for a total replacement of the injured tissue and a perfect reliable approach.^75^ Myocardial tissue engineering approaches, as reported by Chen et al.,^76^ mainly include cell‐based therapy, scaffold‐free cell‐sheet implantation, heart patch implantation, and 3D tissue engineering construction. Cellular‐based therapy employs suspended progenitor or stem cells in saline or culture medium injected into the infarcted area.^77^, ^78^ However, cell survival is disappointing on this occasion regarding poor cell adhesion within the infarcted area. This is mainly due to the raised concentration of ROS inhibiting cell adhesion following MI.^79^ Cell‐containing or cell‐free matrices and cardiac patches are approaches in which the task is to mechanically support the infarcted myocardium to prevent dilation and induce regeneration. This has been shown to effectively slow down the remodeling process and scar formation.^80^ Different strategies to regenerate the cardiac are shown in Figure 2.^81^

CMs are the most suitable cells to be delivered in cell‐based therapy.^35^ However, the major hurdles are poor cell integration with native tissue and thus disappointing cell retention rate.^71^ Moreover, the shortage of a reliable cardiac‐specific cell source and ethical issues attributed to fetal or neonatal CMs are also principal hindering issues. Stem and precursor cells favor sources and differentiated cells, yet there are various particular challenges to overcome. Accurate control of cell differentiation or conversion, teratocarcinogenicity, and concerns associated with allogenic sources are formidable limitations.^35^, ^82^ Efficient recruitment of appropriate cell types and selecting a proper substrate to enhance cell retention and integration, as a result, is highly pivotal.

In brief, cell therapy and tissue engineering are seeking induction of regeneration. Accordingly, different demands should be met in order to improve cardiac performance efficiently. Selection of appropriate matrix, composition, microstructure, chemical and mechanical properties, and cell–matrix interaction makes huge differences.^20^ Cell type and the origin, potency, surface markers, combination, population, dispersion, cell–cell contacts, cell signaling, and gene and protein expression are also required to be determined.^83^ Cell cultivation in vitro before transplantation has been demonstrated to be promising compared to direct delivery of precursor cells. Improved cell retention, survival, and integrity are guided by precultivation.^82^, ^84^, ^85^, ^86^, ^87^

**FIGURE 3:**
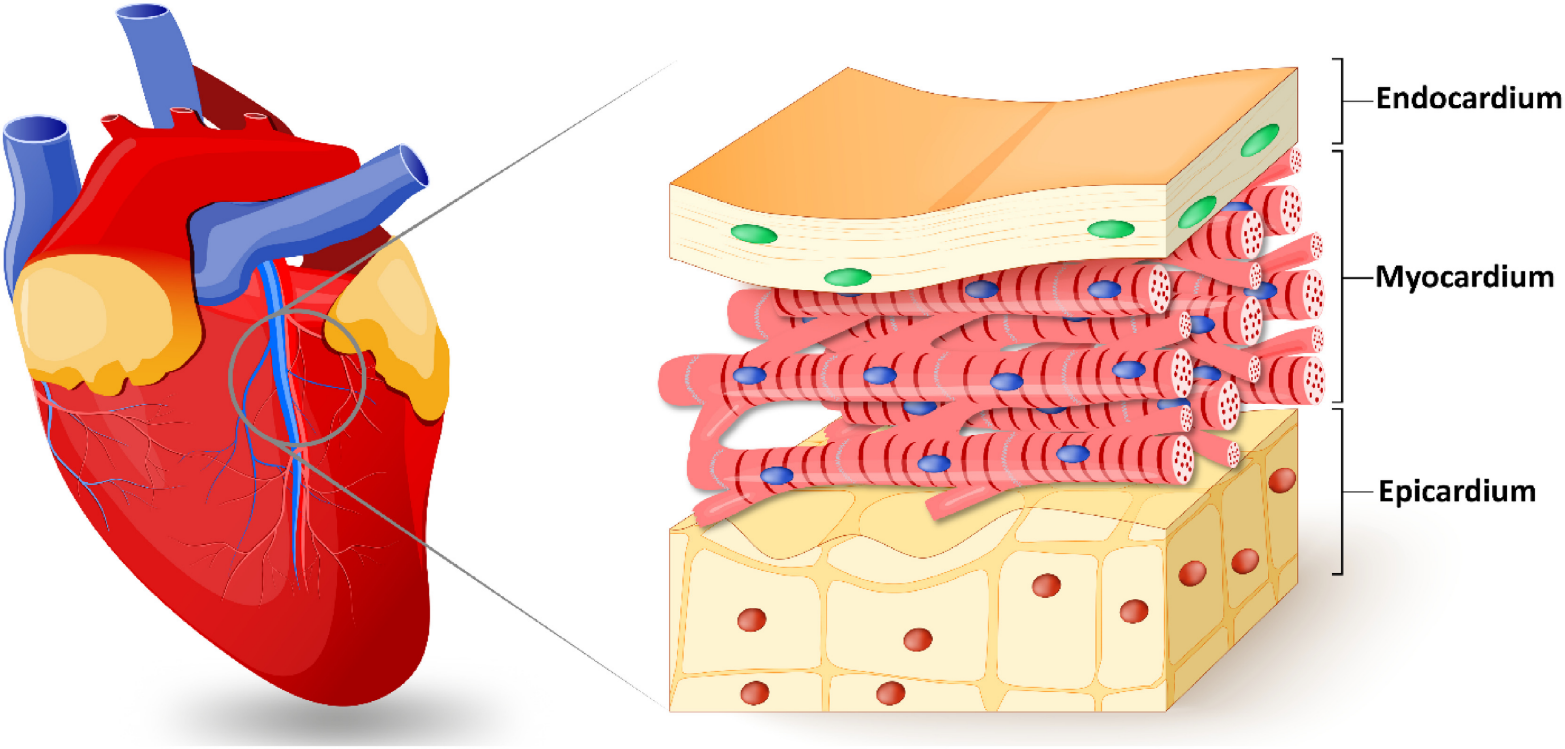
The heart wall structure includes the endocardium, epicardium, and myocardium. Reprinted with permission from Reference 89

Designing a proper structure for cardiac regeneration requires profound knowledge about the cardiac structure, function, and interaction with biomaterials.^88^ The heart is composed of four chambers dividing into ventricles and atria encased in the pericardium. Deoxygenated blood is collected in the right atrium and then is passed through to the right ventricle. Once oxygenation of the blood is completed by contracting and pumping through the lung, it will be collected in the left atrium and takes the way toward the right ventricle. The wall of the heart (Figure 3) includes three strata: the endocardium, epicardium, and myocardium. The interlayer is the endocardium that lies between ventricular and atrial. The myocardium is the middle layer composed of the muscular component of the heart wall. It is dense lamellar, vascularized, oriented, interwoven within collagen, and conductive. The outermost layer is the epicardium.^90^, ^91^ A heart pacemaker is a sinoatrial (SA) node, a small bunch of node cells with a high intrinsic depolarization rate. It lies between the myocardium and the epicardium, juxtaposing the right atrium. Such a node generates the electrical current and sinus rhythm, which contracts the heart and establishes the normal cardiac rhythm, the most mysterious part of heart mechanics. SA, by the aid of internodal pathways (IP), spreads impulses throughout the atria. Three bands of IP, including anterior, middle, and posterior, are conducted in juxtaposing nodes in 50 ms time intervals in which myocardium contractile cells can deliver an impulse to the atrioventricular node using a cell‐by‐cell pathway.^19^, ^43^, ^92^, ^93^ Moreover, impulse straightly is conducted from the right atrium to the left atrium using Bachmann's bundle. By reaching the impulse to the atrioventricular septum, the spreading of the impulse to the myocardial cells is inhibited by the connective tissue of the cardiac skeleton. Na^+^, K^+^, and Ca^2+^ play essential roles in generating the action potential (electrical impulse). Available sodium channels on conductive cells result in gentle sodium ion flux, which causes to ascend the membrane potential from −60 mV to −40 mV. Such movement of ions causes automatic depolarization. After that, the Ca^2+^ gate opens, and ions enter the cell and depolarize to reach +5 mV. Then, repolarization happens by opening the K^+^ channels and closing the Ca^2+^ channels whose membrane potential reaches −60 mV.^94^, ^95^

Wide ranges of biomaterials have been utilized so far to mimic the physiochemical properties of cardiac tissue. Table 1 presents the literature reports on the scaffolds applied in cardiac tissue engineering. It can be seen that all of them showed significant disadvantages despite the seemingly very promising properties. One of the flaws was the lack of conductivity, so these materials cannot substitute myocardium. Therefore, conductive biomaterials have received substantial importance thanks to their inherent feature that recapitulates the cardiac tissue characteristics.^105^, ^106^ CMs' functionality is improved using conductive substrates (with and without electrical stimulation) because of the cardiac synchronizing.^107^, ^108^

**TABLE 1:** Scaffolds utilized in cardiac tissue engineering, their fabrication method, properties, and challenging disadvantages

Material	Fabrication method	Properties	Disadvantages	References
GelMA+Alginate	3D Bioprinting	Controlled anisotropy, seeding endothelialized myocardium, microfibrous hydrogel	Non‐conductive	89
Polyester‐carbon nanotube	Molding	Elastomeric, conductive, degradable, swell‐able	Toxic in a higher amount of CNT	96
Poly(glycerol sebacate)	Microfabrication techniques	Recapitulating cardiac anisotropy, Accordion‐Like Honeycombs, seeded with cultured neonatal rat heart cells, elastomeric, promoting aligned heart cells	Non‐conductive	97
Chitosan/silk fibroin	Layer‐by‐layer electrospinning	Seeded with adipose tissue‐derived mesenchymal stem cells, nontoxicity, biodegradability, anti‐inflammatory, high cohesive strength, hydrophilic nature	Non‐conductive	98
Silk–polypyrrole	Molding	Biocompatible, stable, electroconductive	Without elasticity	99
Graphene–polyethylene glycol	Molding	Anisotropic electrical conductivity	Without elasticity	100
Poly(l‐lactic acid)/ polyaniline	Electrospinning	Enhanced conductivity, good cell viability, and promoting effect on differentiation	Without elasticity	101
Pericardial matrix/CNT	Decellularization/dispersion	Injectable, thermoresponsive	Without elasticity	102
Polyethylene glycol/gold	Crosslinking	Improvement in cellular differentiation	Without elasticity	103
Aniline pentamer‐modified polyurethane/PCL	Blending/porogen leaching	Conductivity supported neonatal cardiomyocytes (CMs) adhesion and growth	Phase separation	104

**FIGURE 4:**
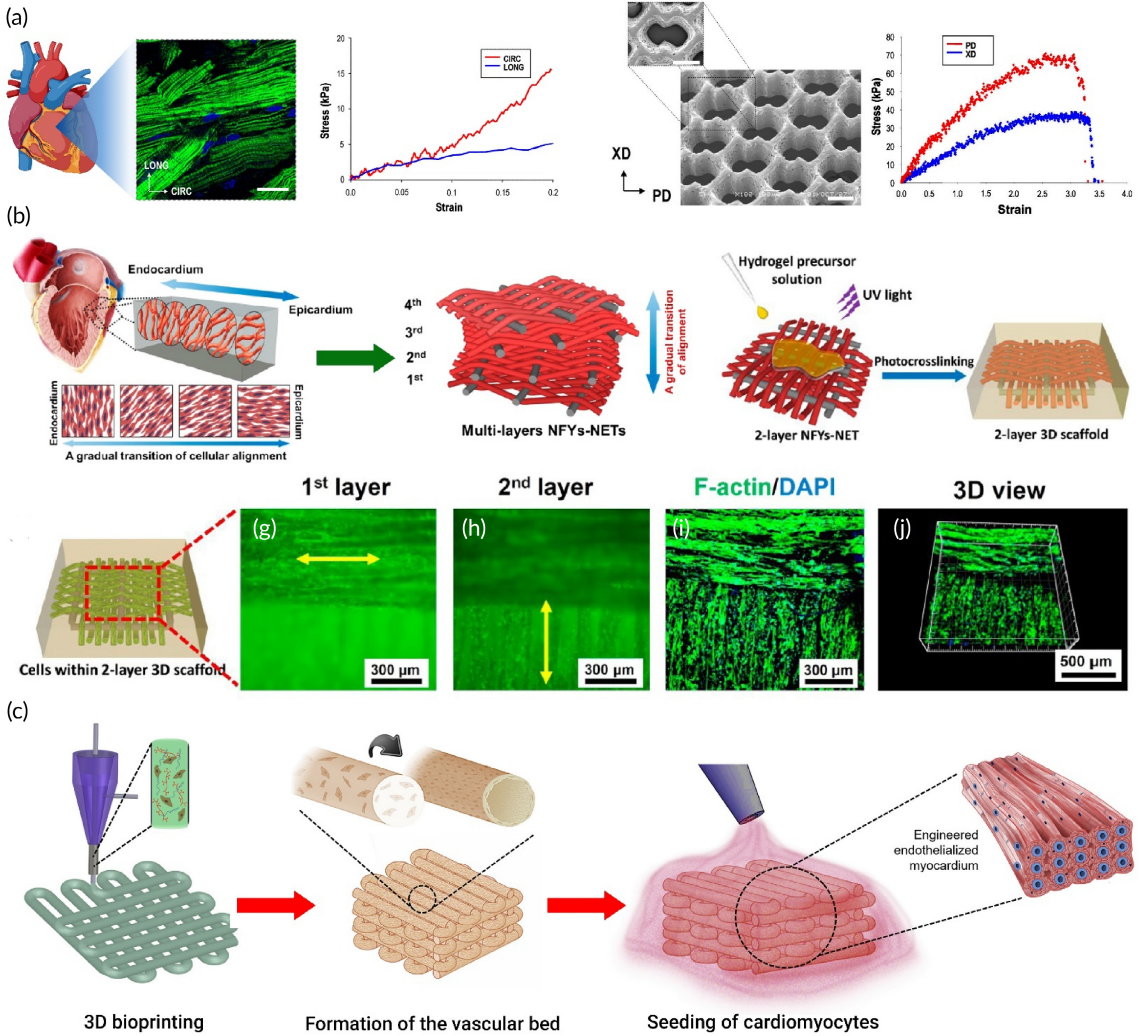
(a) Accordion‐like honeycomb scaffold that can mimic the naïve heart structure. Reproduced from^97^ with permission from Nature (b) Interwoven scaffold composed of fiber embedded in a hydrogel which can recapitulate the heart structure. Reproduced from^113^ with permission from the American chemical society (c) 3D printed scaffold which can mimic the cardiac structure and is capable of cell seeding. Reproduced from^89^ permission from Elsevier

In this regard, the architected scaffold should recapitulate the 3D anisotropic structure of the heart to provide a proper milieu for cellular activity. Bundling the undulated fiber of perimysial collagen inside the honeycomb‐like structure forms an endomysial collagen layer that surrounds the cardiac muscle fibers.^109^, ^110^ Such structure endows the anisotropic features with mechanical and electrical characteristics. Various classes of the 3D structure have been designed over the years to mimic the heart function by taking credit for maximum cellular activity. For instance, an accordion‐like honeycomb structure with elastomeric properties was utilized for recapitulating cardiac function.^111^, ^112^ Engelmayr et al. seeded the neonatal cardiac cells on structures exhibiting similar mechanical properties to the right ventricular myocardium. Moreover, the electric field can contract the heart cell, and the cell alignment was higher than the control state (Figure 4a).^97^ Wu et al. presented a 3D scaffold composed of carbon nanotube conductive fibers embedded in a hydrogel matrix (Figure 4b) capable of mimicking the anisotropic properties of cardiac muscle along with providing a proper conductivity. The designed scaffold exhibited proper biocompatibility, cell orientation, and enhanced CMs' maturation.^113^ Ys et al. printed a 3D scaffold (Figure 4c) with controlled anisotropy capable of printing the cells within the hydrogel bio‐ink. Developing such a method can open a bright horizon to fabricate biomimetic scaffolds.^89^, ^114^, ^115^

**FIGURE 5:**
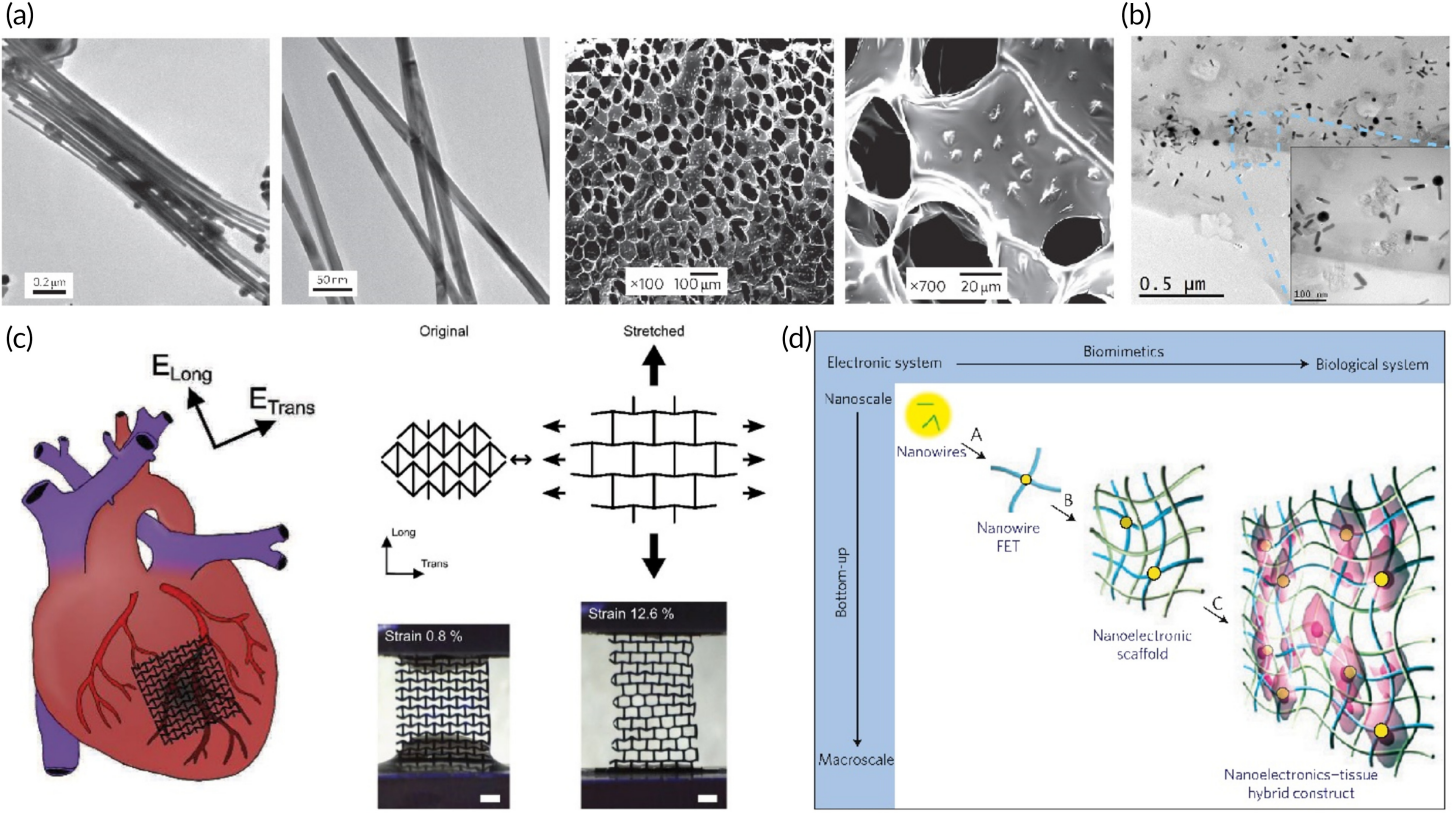
Engineering of electrically conductive scaffolds. (a) Gold nanowires act as conductive bridges when embedded in macroporous alginate hydrogels to allow better electrical signal propagation and contractile behavior of CMs. Reproduced from Reference 116 with permission from Nature. (b) TEM micrographs showing gold nanorods distribution within a thin layer of GelMA hybrid hydrogel (1.5 mg/ml). Reproduced from Reference 117 with permission from Elsevier. (c) A conductive patch that has an auxetic design and can mimic the anisotropy of the myocardium. Reproduced from Reference 118 with permission from Wiley. (d) Nanoelectronics integrated into cardiac tissue allows spatiotemporal electrical signal propagation. Reproduced from Reference 119 with permission from Nature

Concluding, electrical conductivity is a crucial factor in scaffolds fabricated for cardiac tissue engineering. Due to the specific electrical properties of cardiac tissue, in which contractility is the result, the electrical conductivity of the construct, signal propagation, and synchronous contraction capability also merit consideration (Figure 5). Electroactivity is defined in further detail within the next part.

## 3 ELECTRICAL CONDUCTIVITY

**FIGURE 6:**
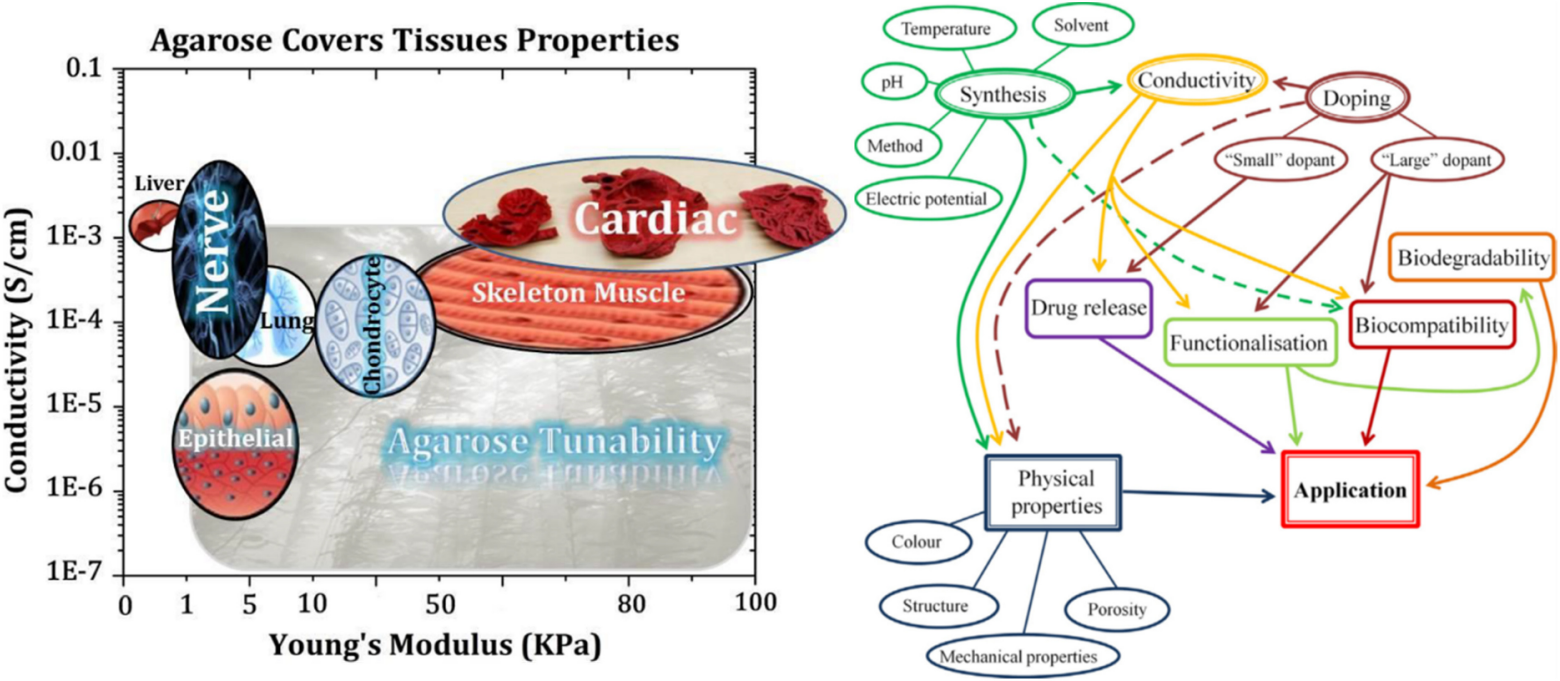
The conducive platform's properties are adjustable with various tissues. The plot on the left‐hand side advises on the selection of biomaterials for a target tissue considering their conductivity and mechanical properties, while the right‐hand one provides the investigator with a quick view of the microstructure–property–performance relationship when one takes the first step in the selection of conductive biomaterials for tissue engineering and regenerative medicine uses.^129^

In an attempt to tissue repair and regeneration, engineered constructs mimic the original niche through their specific features.^120^, ^121^ This requires an appropriate combination of the designed construct with particular mechanical, physiological, and electrical properties, similar to the native tissue. Since any communication, including scaffold and cell receptors' interaction, cell–cell signaling, and intracellular activities, is disposed to be engaged in a compelling performance, electroactive substrates in which cells are seeded for tissue engineering efficiently promote cell behaviors and regeneration.^122^, ^123^, ^124^, ^125^, ^126^ A specific voltage across cell membranes specifies the resting potential and ion exchange and varies depending on cell type and tissue.^127^, ^128^ Hence, regulation of the ion exchange highly impacts cell behaviors, including cell attachment, cell proliferation, protein expression within cells, and cell maturation. Less resting potential through cell membranes induces more proliferative capacity, as observed in cancer and stem cells.^127^ Thus, an appropriate conductivity of the designed construct in tissue engineering regulates ion transfer resulting in enhanced cell proliferation.^127^ Figure 6 shows that conductive biomaterials, according to adaptability, can be designed in order to target tissue to improve regeneration. Substrate conductivity, which can be accustomed by synthesis assay, can affect drug release design, and cell behavior.^129^

Bone tissue regeneration, for instance, is electricity attributing. Applied mechanical forces to bones induce an electrical field owing to the piezoelectricity characteristic.^130^, ^131^ Apparently, electrical stimulations induce cell proliferation and bone healing.^132^, ^133^, ^134^ Promoted bio‐mineralization, accelerated formation of tri‐calcium phosphate, improved cell proliferation, and osteogenic differentiation has been observed in conductive bone matrices in contrast to nonconductive ones.^135^, ^136^

Conductivity is also recommended regarding neural and cardiac tissue regeneration.^125^ Data transfer is conducted by an action potential within neural networks, requiring a conductive substrate.^137^, ^138^ Likewise, upregulated expression of neural progenitor markers, enhanced cell differentiation toward neurons, and promoted neural induction within conducted substrates have been demonstrated.^139^, ^140^, ^141^, ^142^, ^143^ Other than neural tissue, muscles' contraction is also followed by an electrical signal propagating throughout the tissue. In cardiac tissue repair, the conductive substrate is in charge of electromechanical and electrochemical transmittance leading to electrical stimulation of cells. Synchronized contractions are attainable as long as the propagation of electrical impulses is achieved.^144^, ^145^ It has also been demonstrated that conductivity in cardiac tissue engineering modulates cellular function and enhances cardiac gene expression.^60^, ^146^, ^147^

In contrast to skeletal muscles in which contractions are neurogenic, smooth and cardiac muscle contractions are myogenic, initiating from the heart itself, along with a rhythmic and autonomous behavior. Contractions within cardiac muscle generally arise from impulses generated by the sinoatrial (SA) node located in the wall of the superior vena cava. The atrioventricular (AV) node, positioned in the atrial region of the septum, is the next spot generating impulses which are later spread through the ventricular walls via the atrioventricular bundle (bundle of His), targeting Purkinje fibers. Finally, contractions occur initiating from the apex of the heart and spreading upward through the walls of the ventricles. This order is likely because Purkinje fibers originate from the inner ventricular septum and extend to the papillary muscles toward the ventricles' walls.

As indicated, cardiac muscle conductivity is mainly attributed to the conducting Purkinje fibers,^129^ nodal cells, and fibers of the bundle of His, altogether known as autorhythmic or conducting cells of the heart. They possess specific characteristics apart from CMs, facilitating cardiac‐specific action potential to be transmitted. On the other hand, contractility is mainly ascribed to CMs, termed as working or contractile cells. In brief, CMs contraction is enabled owing to the conducting cells inducing contractile stimuli through transmitting action potentials to all cells.^148^, ^149^, ^150^

CMs are joined together via intercalated discs. Probing them, particular structures together are involved, including fascia adherens, desmosomes, and gap junctions. Gap junctions are in charge of the ion diffusion through channels and action potential allowance through cell membranes. At the time of contractions, due to consecutive cell membrane depolarization, impulses are directly spread through the atriums over gap junctions. Connexins, as constructing gap junction proteins, create channels through which ions can pass among adjacent cells. Other types of cell junctions within intercalated discs, on the other hand, are basically responsible for supporting CMs since relatively high mechanical forces as a result of constant contractions are applied to them. CMs are adequately bound and held together via these structures.^151^, ^152^

Interruptions through intercalated discs thus uncoupled CMs and disrupted contractions, as already defined previously, are one of the main complications of MI.^153^ Efficient treatments to the ischemic myocardium approaching tissue regeneration have to meet different demands, among which electrical conduction has been widely studied. Due to the heart muscle anatomical structure, an anisotropic, discontinuous electrical conduction^154^ is reported to match the amount of 1.6 × 10^−3^ S cm^−1^ along and 5 × 10^−5^ S cm^−1^ across the myocardium.^138^, ^155^, ^156^, ^157^, ^158^ Studies have furthermore confirmed the influence of electrical conduction and stimulations on the regenerative behavior of body tissues. Cell division, tissue growth, and wound healing, as evidenced by studies, are observed to be obviously affected.^159^

Electrical conductivity comes after moving ions, carrying charge in one or more directions within the substance. It is provided by the flow of negatively charged electrons and positively charged holes. Seeking tissue regeneration, conductive materials, or incorporated electroactive particles and other materials are employed to promote electroactivity. Conductive polymers, metallic nanoparticles, and carbon‐based materials are currently the standard choices in this field. Depending on the material selected and the application, optimization is always necessary to reach good electrical, mechanical, and biological properties.^160^

Some polymers require a doping process in order to be modified as conductive materials.^144^ Predominant conductive polymers used in tissue engineering include polypyrrole (PPy),^161^, ^162^ polyaniline (PAni),^163^, ^164^, ^165^ polythiophene (PTH), and its derivatives.^166^, ^167^, ^168^, ^169^ Apart from their proper conductivity, the use of conductive polymers bears disparate advantages, including producibility, processability, surface modification potency, relatively low cost, and suitable biocompatibility. Meanwhile, comparatively poor solubility and challenging biodegradability demand further consideration.^160^, ^162^ In cardiac tissue engineering specifically, a close elasticity resemblance to the native myocardium is essential owing to the frequent contractions of the heart. Polymers are likely to exhibit an undesired rigidity which makes their use limited in this field.^155^, ^158^

Other than conductive polymers, prevalent metallic nanoparticles widely used in biomedicine are gold,^170^, ^171^, ^172^, ^173^, ^174^ copper,^175^, ^176^ and silver^177^, ^178^, ^179^, ^180^ nanostructures. High electrical conductivity, high surface‐to‐volume ratio, ease of synthesis, and magnetic and antibacterial properties have disposed of metallic nanoparticles to be engaged in the area of tissue engineering and regenerative medicine. Contrastively in a long‐term spectrum, cytotoxicity is the foremost hurdle making biocompatibility of these materials a severe challenge.^180^, ^181^

In addition to the materials mentioned above, carbon‐based materials also have the magnificent potential to result in electroactivity. These include graphite,^182^, ^183^ graphene,^184^, ^185^, ^186^ graphene oxide,^187^, ^188^ reduced graphene oxide,^189^, ^190^, ^191^ carbon nanofibers,^60^, ^192^, ^193^, ^194^ carbon nanotubes,^156^, ^195^, ^196^ fullerene,^197^, ^198^ carbon quantum dots,^199^, ^200^, ^201^ and nanodiamonds.^202^, ^203^, ^204^ Particular mechanical, electrical, thermal, and optical properties bring about the opportunity for carbon‐based nanomaterials to be involved in the field of tissue repair.^205^, ^206^ Conductive carbon‐based polymers, as well as carbon‐based nanomaterials and their application in developing electro‐active cardiac tissues, are discussed in detail in the following parts.

## 4 CONDUCTIVE POLYMERS

**FIGURE 7:**
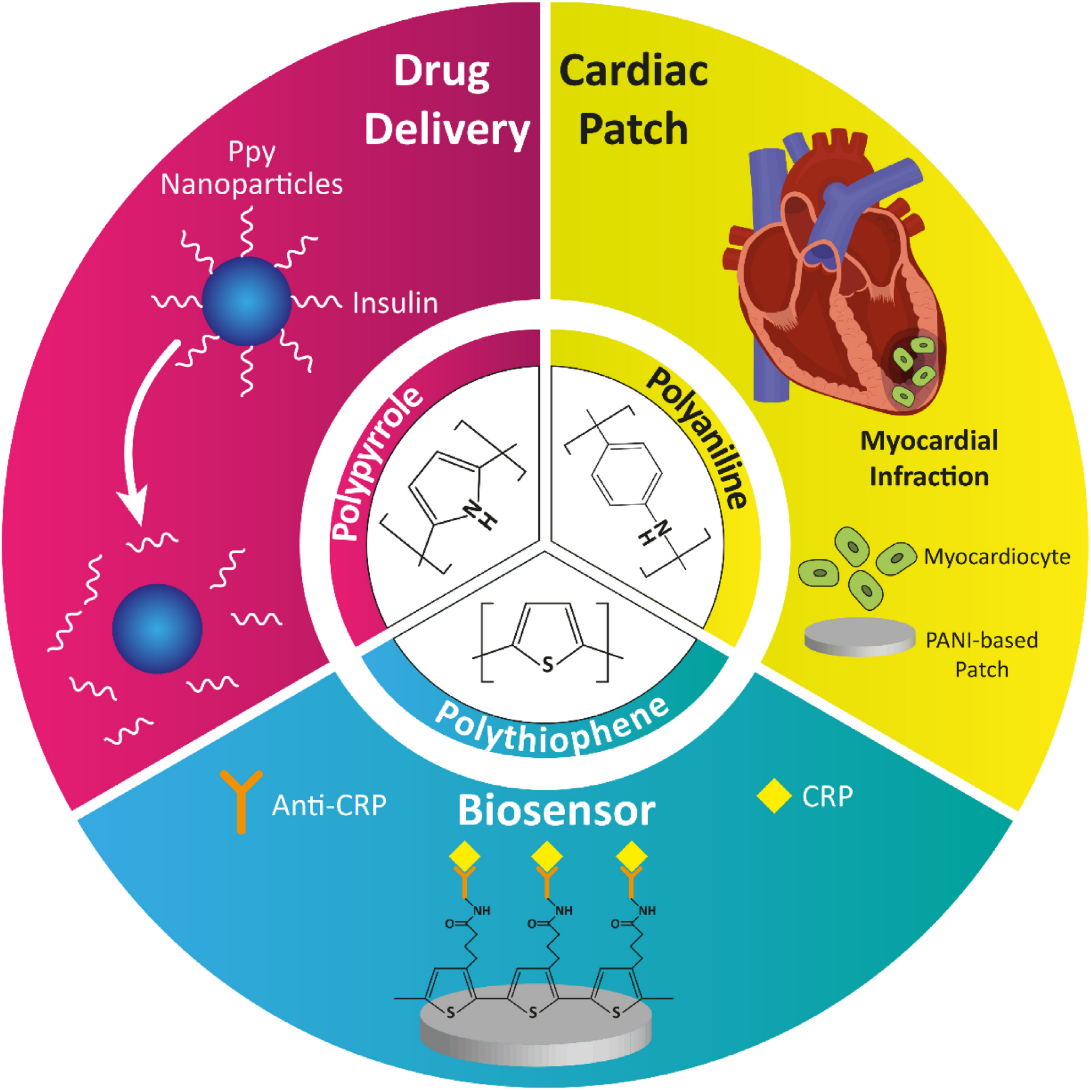
Application of conductive polymers in nanomedicine

The simplest method to provide the electroactivity of scaffolds and other materials used in cardiac tissue engineering, which should be characterized by an efficient cellular response, is the application of conductive polymers.^207^, ^208^ In general, conductive polymers mainly include polypyrrole.^209^ polythiophene, and the most well‐known member of this family, polyaniline (PANI).^210^ These polymers have shown promising features for the regeneration of electrically responsive tissues.^90^ The conductivity mechanism of inherently conductive polymers is ascribed to the sequential *sp* ^2^ hybridized carbon existing in their structure.^211^ Combination of Pz orbital with residual valence electron results in delocalized orbitals allowing electrons to move freely during the doping process. Oxidative and reductive doping yields *p*‐type and *n*‐type conductive/semi‐conductive materials. Typically, the conductivity of such polymers can be tuned from 10^−6^ to 10^2^ S/cm. The primary cell functions, such as attachment, proliferation, migration, and differentiation, could be modulated through electrical stimulation.^212^ Figure 7 displays an overall view of conductive polymers' usage in nanomedicine. Some successful attempts can be found in well‐established reports devoted to neural,^213^ bone,^214^ skin,^215^ and more specifically, cardiac tissues.^216^ However, there have been confusing reports on the biocompatibility function of conductive polymers and the cytotoxic characteristics of such materials.^147^

Polypyrrole is one of the favorable conductive polymers widely utilized as a biomaterial.^49^ Oxidation of the pyrrole produces a conjugated polymer chain with a positive charge. Easy and flexible synthesis in large quantities at room temperature in a wide range of solvents, fabrication with a large surface area with different porosities, and easy modification of this polymer are advantages that make it more suitable for biomedical applications through the incorporation of bioactive molecules. Biodopants and anionic molecules have been utilized to balance the polymer charge. Polypyrrole at the oxidized level needs anionic dopants as a complement to neutralize the positively charged backbone. The redox state of the polypyrrole exhibits a substantial effect on protein adsorption and desorption following the cellular attraction. One of the practical problems that researchers are faced with in vivo analyses, is the low adhesiveness of the scaffold to the tissue. In this regard, Liang et al.^217^ synthesized an adhesive as a self‐healing injectable hydrogel patch to regenerate the myocardium defect. Dopamine incorporated in hydrogel structure endowed the adhesiveness to the hydrogel and enhanced hydrogel performance.

The other group of conductive polymers includes polythiophene and its derivatives. An absorbing conjugated polymer is a poly(3,4‐ethylenedioxythiophene) (PEDOT), polythiophene (PTh) derivative that is formed by the polymerization of the bicyclic monomer 3,4‐ethylenedioxythiophene. This polymer possesses good electrical, chemical, and environmental stability and better conductivity and thermal stability than PPy. Another PTh derivate of interest is poly(3‐hexylthiophene) (PHT). PHT has good solubility in organic solvents, excellent environmental stability, and electrical conductivity.^218^ The percentage of conductive polymer should be optimized in conductive scaffolds because high contents of conductive polymer could result in toxicity. Spencer et al. synthesized gelatin poly(3,4‐ethylenedioxythiophene) polystyrenesulfonate (PEDOT:PSS) hydrogel, which supports the C2C12 myoblasts. According to their report, the percentage of PEDOT should be 0.1 to exhibit the optimum performance of hydrogel.^219^

PANI is the most utilized conductive polymer in biomedical applications. Recently, aniline oligomers have attracted massive attention due to their proper processability, biocompatibility, degradability, and ease of synthesis.^220^ PANI and its oligomers have been utilized to synthesize engineered conductive biocompatible polymers like injectable hydrogels for tissue engineering,^217^ drug/gene delivery,^221^, ^222^ and wound dressing.^165^, ^223^ In this sense, research directed at the usage of PANI in cardiac regenerative nanomedicine was stressed in this part. Table 2 summarizes studies in which PANI was examined for cardiac tissue engineering.

**TABLE 2:** PANI‐based biomaterials applied in cardiac tissue engineering applications

Biomaterials	Cells	Main Results	In vitro/in vivo	References
PANI/Polyethersulfone	Cardiovascular disease‐specific induced pluripotent stem cells	Increased cell number	+/−	^224^
PANI/PLGA	CMs	Enhanced cell adhesion	+/−	^225^
Tetra‐aniline/PEG	C2C12 Myoblast	Enhanced MI regeneration	+/−	^226^
Tetra‐aniline/PEG/hyaluronic acid	Adipose‐derived stem cells (ADSCs)	Enhanced endothelial and muscle cell homing, enhanced MI regeneration	−/+	^227^
PU/siloxane/tetra aniline	CMs	Enhanced cell function even without external electrical stimulation	+/−	^228^
PANI/PLA	C cell	Enhanced cell viability and proliferation	+/−	^101^
PCL‐aniline trimer	C2C12 Myoblast	Enhanced cell proliferation and myogenic differentiation	+/−	^229^
Poly(citric acid‐copolycaprolactone)‐aniline hexamer	C2C12 Myoblast	Excellent cytocompatibility	+/+	^230^
Terta aniline‐ poly(n‐iso propyl acrylamide)	H9c2 cells (rat cardiac myoblast)	High cell viability	+/+	^231^
Polyurethane–aniline pentamer/PCL	CMs	Enhanced cell proliferation and adhesion, proper cardiac gene expression	+/−	^104^, ^163^

**FIGURE 8:**
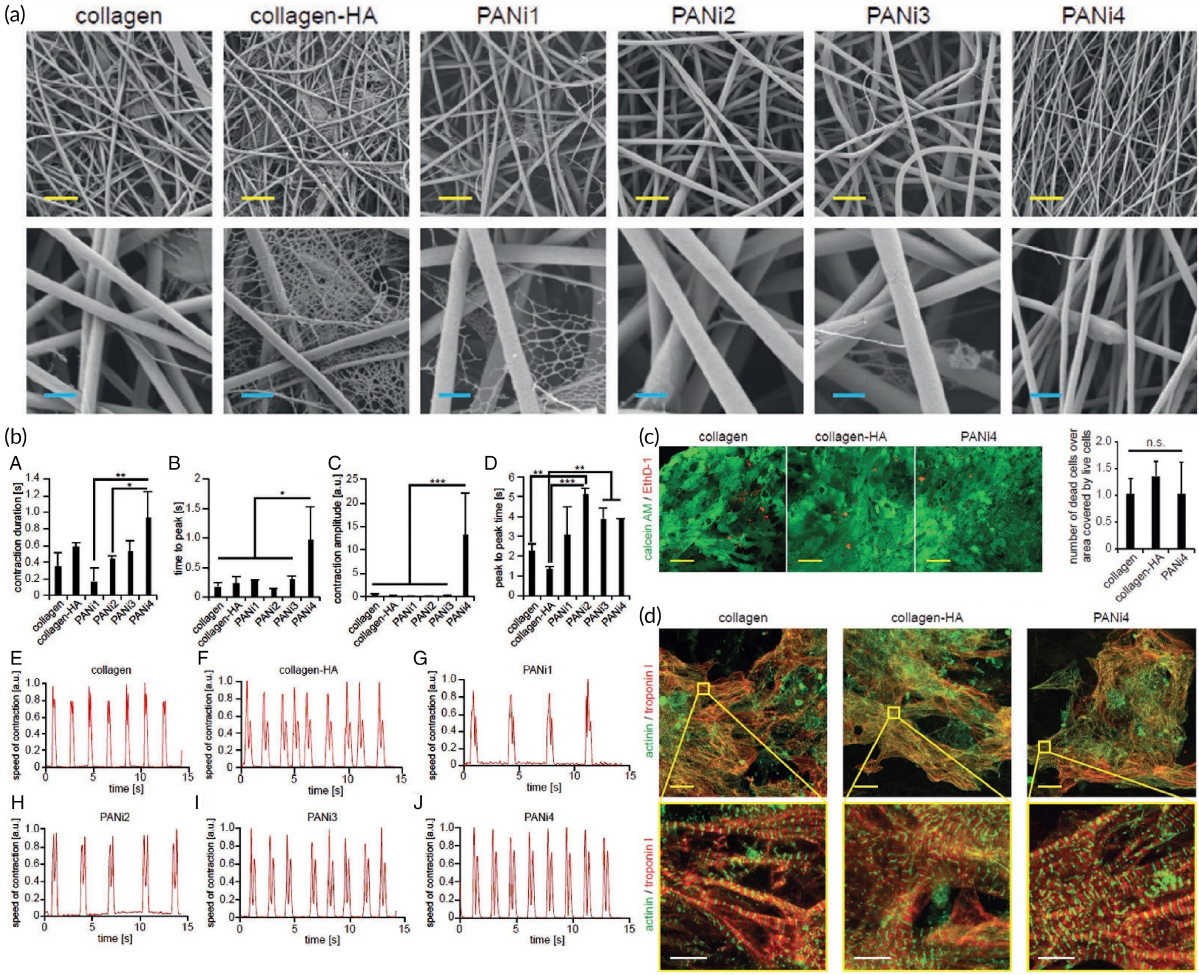
Nanofibrous composites containing polyaniline, collagen, and/or hyaluronic acid for cardiac tissue engineering. (a) SEM images of electrospun fiber mats. (b) Musclemotion analysis of the beating behavior of CMs on different fiber mats indicates that PANi4 fiber mats demonstrate the most desirable characteristics for cardiac tissue engineering. (c) The viability of neonatal rat cardiac cells cultured on electrospun fiber mats at day 5 based on live and dead staining exhibited no difference in the viability of hiPSC‐derived CMs on either scaffold. (d) confocal images of hiPSC‐derived CMs cultured on electrospun fiber mats at day 5 stained for the CM‐specific markers troponin I and/or sarcomeric‐α‐actinin. Reproduced from Reference 237 with permission from Wiley

Oligoaniline segments have shown different effects on cells depending on their end groups and molecular weights. Carboxylic acid end‐capped aniline pentamer exhibited appropriate biocompatibility, cell adhesion, proliferation, and growth compared to the other oligomers.^138^, ^232^, ^233^ Moreover, its hydrophobic nature allows self‐assembly in the biological milieu, promising for the developing a drug delivery vehicle.^234^ Dong et al.^226^ synthesized a self‐healing injectable conductive hydrogel based on tetra‐aniline and polyethylene glycol (PEG), which could carry the C2C12 and H9c2 cells. In vitro and in vivo subcutaneous injection revealed that such a platform could preserve cells during and after injection leading to enhancement of the myocardial infarction regeneration. Heart contraction takes mainly origins in reflux problem. Contraction of the heart squeezes the excess blood out of the heart. To resolve this situation, it is necessary to use adhesive materials to maintain heart performance at normal level during operational conditions to avoid scaffold detachment. Conductive elastomers based on aniline oligomer as a hard segment and polycaprolactone (PCL) as a soft segment were the subject of a study. The role of the addition of PEG to the scaffolds and nanostructures has been investigated widely, even in the Ti_3_C_2_T_x_ (MXene)‐based nanomaterials. In this manner, PEG addition to the electrically conductive MXene has increased the relative cell viability, and in this regard, iCMs cells were seeded on the substrate based on MXene decorated with PEG, and the results showed considerable Connexin43 (CX43) expression. Besides, the free‐PEG structure based on MXene showed less sarcomere length in the alpha‐actinin structure compared to the PEG‐coated MXene nanostructures.^235^ It was demonstrated that the aniline oligomer enhances cell proliferation and adhesion of C2C12 myoblasts. Such scaffolds can support neonatal CMs' growth and adhesion along with the expression of cardiac genes such as cytoskeleton alignment (actinin‐4) and muscle contraction and relaxation (troponin‐T) genes.^104^, ^228^ In a study by Mawad and co‐workers, a dopant was immobilized in the conductive scaffold. Prefabricated chitosan film was used to facilitate polymerization of aniline in the presence of a small multivalent dopant, phytic acid, which attributed to a new approach for crosslinking the multivalent anionic dopant to the PANI patch. This patch was applied to a skinny slice of cardiac tissue and the whole heart as well. By the adrenaline injection, the results revealed that these photoadhesion conductive patches do not influence the proarrhythmic state of the heart under stress; therefore, they are safe for cardiac application.^236^ According to experiments, polylactic acid (PLA)/PANI nanofibrous sheets show good cell viability and proliferation, similar to PLA nanofibrous sheets. The myotubes on the PLA/PANI sheets are longer and more mature than those on the PLA sheets. CMs grown on PLA/PANI nanofibrous sheets show more synchronous beating with a much higher rate than PLA nanofibrous sheets. Moreover, there are more synchronized calcium transients in PLA/PANI groups than in PLA groups (Figure 8).^101^

**FIGURE 9:**
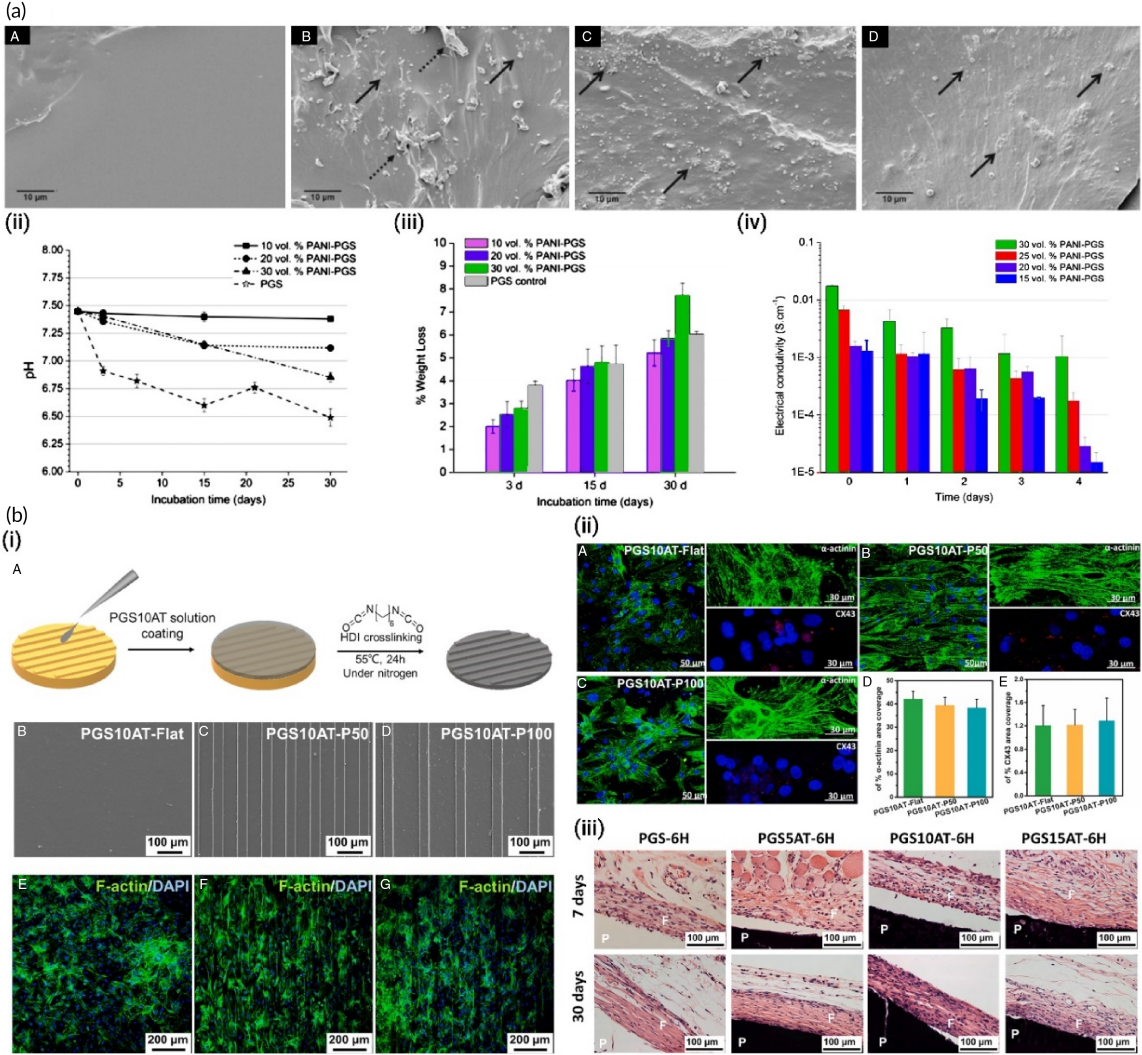
(a) Conductive polyaniline (PANI)–poly(glycerol sebacate) (PGS) composites for cardiac tissue engineering. (i) Representative SEM images showing cross‐section views of (A) pure PGS, (B) 10 vol% PANI–PGS, (C) 20 vol% PANI–PGS, and (D) 30 vol% PANI–PGS samples. Dashed arrows in (b) point to polymer matrix sheared during sample preparation. (ii) pH Variance in a 30 days period of in vitro degradation in PBS medium for pure PGS, and 10, 20, and 30 vol% PANI–PGS composites. (iii) Weight loss percentage of pure PGS, and 10, 20, and 30 vol% PANI–PGS composites due to in vitro degradation in PBS medium. (iv) Electrical conductivity alterations of 15, 20, 25, and 30 vol% PANI–PGS composites. Reproduced from Reference 147 with permission from Elsevier. (b) Micropatterned electroconductive poly(glycerol sebacate)‐aniline scaffolds for cardiac tissue engineering. (i) (A) schematic illustration of the preparation of micropatterned PGS10AT‐6H films. Representative SEM images of (B) PGS10AT‐Flat, (C) PGS10AT‐P50, and (D) PGS10AT‐P100 films. The immunofluorescence staining images show the F‐actin (green) and nuclei (blue) of CMs on (E) PGS10AT‐Flat, (F) PGS10AT‐P50, and (G) PGS10AT‐P100 films after culture for 2 days. (ii) The immunofluorescence staining images of the expression of cardiac‐specific markers (α‐actinin (green) and Cx‐43 (red)) on (A) PGS10AT‐Flat, (B) PGS10ATP50, and (C) PGS10AT‐P100 films after 8 days cultivation. (iii) Histological evaluation of subcutaneously implanted polymer films. Reproduced with permission from Reference 242, Elsevier

Despite all of the advantages of conductive polymers, there have been confusing reports on their biocompatibility (mainly PANI) and the cytotoxic characteristics.^147^ Such contradictory actions of conductive biomaterials in affecting cardiac cell function have made investigations challenging. It was reported that PANI plays a dual role in cardiac regeneration, which results in limited practical use, mainly arising from poor solubility in common organic solvents, low processability, nondegradability under the physiological conditions, and inflammatory response because of the de‐doping process.^238^ Dopant concentration/type, reaction time, functional groups, and molecular weight of PANI and oligoanilines exhibit an important effect on biocompatibility and performance.^120^, ^239^ Bidez et al. evaluated the adhesion and proliferation of H9c2 cardiac myoblasts on both the non‐conductive emeraldine base (PANI) and its conductive salt (E‐PANI) (doped with 1 M HCl) of PANI thin films.^240^ Upon 15 min early‐stage incubation, H9c2 cells could adhere to both PANI and E‐PANI in an almost similar manner, signifying a 7% fall in initial adhesion for tissue culture plate (TCP). H9c2 cells were seeded at a low density of 104 cells/cm^2^ and proliferated up to 200 h. Relative cell numbers on the E‐PANI substrate showed a significantly extended lag phase of growth compared to PANI and TCP, attributed to the leakage of residual acid dopants, whatever the extensive wash after doping. Interestingly, after 100 h of dopant dissipation, the doubling time of the cells on the E‐PANI surface was significantly shorter (54 ± 11 h) than that of cells growing on PANI (78 ± 20 h) and TCP controls (93 ± 25 h). In addition, cell numbers on E‐PANI were equal to those on TCP at 200 h post‐seeding. As a matter of confusion, the authors mentioned that these findings could not be generalized to all cell types, suggesting that the biocompatibility of conductive polymers might be restricted from cell to cell. Morphological and cytoskeletal architectural analyses of H9c2 cells grown on different substrates by rhodamine‐phalloidin staining confirmed no difference in the cytoskeletal architecture or histotypic morphology after 144 h. Physical parameters of the films, including the thickness and surface roughness, were taken as key factors affecting the cell‐surface interaction. Moreover, it was reported that E‐PANI, when incubated in Dulbecco's Modified Eagle's Medium (DMEM) at 37°C, retained a significant level of electrical conductivity for at least 100 h. Later on, this group conducted a similar study to improve the biocompatibility of PANI via covalently attaching biologically active oligopeptides Tyr‐Ile‐Gly‐Ser‐Arg (YIGSR) and a scrambled control sequence Arg‐Tyr‐Ser‐Gly‐Ile (RYSGI) to PANI to enhance cell attachment, proliferation, and differentiation for neuronal and cardiac tissue engineering.^241^ PC‐12 pheochromocytoma cells exhibited limited adhesion and proliferation on untreated PANI. The biocompatibility of PANI films to PC‐12 improved by grafting adhesive peptides or forming electroactive complexes of PANI with natural polymers such as collagen. Biocompatibility assessment of thin films of PANI was performed with H9c2 rat cardiac myoblast cells in both conductive and nonconductive forms. Although cells on the E‐PANI substrate exhibited a significantly extended lag phase of growth compared to the PANI and tissue‐culture‐treated polystyrene (TCPS) in 100 h, the doubling time of the cells on the E‐PANI surface was significantly shorter (55 h) than that of cells growing on PANI (78 h) and TCPS controls (90 h). The cells grew more slowly initially on E‐PANI but eventually caught up, and cell numbers were equivalent to those on the TCPS control by 200 h. The experimental data suggested that conductive PANI, when maintained in an aqueous physiologic environment, retained a significant level of electrical conductivity for at least 100 h, even though this conductivity was decreasing over time due to the partial de‐doping with the culture medium. Qazi et al.^147^ developed conductive composite films based on PANI blended with poly(glycerol‐sebacate) (PGS) at volume ratios of 10, 20, and 30 PANI via solvent casting. Degradation studies on prepared samples in PBS medium at 37°C showed more weight loss in composites than pure PGS over 30 days. A direct relationship was found between the volume content of PANI in the composites and the weight loss percentage over a specific incubation time, with the highest weight loss, occurring for 30 vol% PANI–PGS composite films (7.72%). One of the issues associated with the degradation of PGS is the formation of acidic by‐products. However, this group mentioned the pH buffering effect of PANI that stops experiencing a sharp drop in pH profiles of the PANI–PGS composites compared with that for pure PGS. It was cited that in composites with higher amounts of PANI, higher amounts of the dopant could leach out following degradation, which causes a drop in pH. The conductivity values of the blended composites based on PANI and PGS were increased by increasing PANI volume content by 30 vol% showing the conductivity of 1.03 × 10^−3^ S/cm. Over 4 days, the conductivities decreased with time but did not fall below more than an order of magnitude for any composite samples (Figure 9a). Roberts‐Thomson et al.^243^ designed a patch to be assigned to the epicardium. Representative stress–strain curves of electroactive composites showed a direct relationship with the amount of PANI. Although the addition of PANI could improve the deformation behavior, no direct relationship has been recorded for this mechanical property. There are confusing reports on increasing the amount of PANI on the mechanical properties of the resulting composites. Li et al.^244^ also investigated the potential application of electrospun PANI‐contained gelatin fibers as conductive scaffolds for tissue engineering purposes. Through comparing the mechanical properties of electrospun pure gelatin to PANI‐gelatin blend fiber sheets, it was found that both tensile strength and modulus were increased by increasing the concentration of PANI in the blend solution to a 45:55 ratio of PANI to gelatin which could strengthen the electrospun blend fibers. However, at this concentration, the maximum deformation (elongation) of the scaffolds dropped significantly. The addition of more amounts of PANI also made the PANI‐gelatin blend fibrous scaffolds less elastic. However, Jeong et al. cited that the addition of PANI to blended poly(l‐lactide‐*co*‐ε‐caprolactone) (PLCL) by electrospinning could decrease Young's modulus, tensile strength, and elongation at break.^245^ In contrast, Ghasemi‐Mobarakeh reported increased tensile strength but reduced elongation at break for electrospun PANI mixed with poly (ε‐caprolactone)/gelatin.^246^ Wu et al. demonstrated that the electrical conductivity of substrate exhibited a higher impact on cellular activity than its mechanical properties.^247^ In the embryonic heart, electrical impulses propagate unidirectionally from the sinus venosus and appear to be involved in cardiogenesis. Mohammadi et al. demonstrated that the usage of the unidirectional electrical stimulation to the cells significantly increased the number of cardiac Troponin T (cTnT+) cells in comparison to multidirectional electrical stimulation via random fibrous scaffolds; so that the scaffold could mimic the unidirectional wave of electrical stimulation in the heart.^224^, ^248^ Intracellular ROS production was promoted via electrical pulses, which stimulated the release of intracellular Ca^2+^, underlining its important role in cardiac gene expression and differentiation by enhancing GATA4.^249^ Electrical stimulation can be linked to cardiac gene expression by Calcineurin/NFAT and GATA4 pathways.^250^ Liu et al.^251^ synthesized a biodegradable electroactive hydrogel (AP‐g‐GA) of aniline pentamer (AP) grafting gelatin (GA) by a coupling reaction between the carboxyl group of AP and the amino side group of GA in an aqueous solution. The rigidity of the EM AP made it difficult for the GA chains to freely coil by coiling around the template of the EMAP aggregate. It was found that with increasing the content of AP graft, the degradation rate was decreased. GA lost about 80% of its weight at 28 days; however, AP‐g‐GA polymers did not experience weight loss of more than 65%. The authors stated that the hydrophobicity and steric hindrance of AP‐g‐GA increased by introducing AP to GA, but still, it was considered biodegradable. With increasing the content of AP in AP‐g‐GA copolymer, the cell viability diminished slightly. However, pure EMAP exhibited low cell viability compared to that of GA and AP‐g‐GA. The improvement in cytocompatibility of the AP‐g‐GA was ascribed to the biocompatibility of the gelatin. The degradation products of AP‐g‐GA also showed no cytotoxicity with a slight decrease when the concentration was 50 mg ml^−1^. It was stated that the introduction of more AP in the structure of copolymer could increase the charge and the toxicity at the same time. While electroactivity could accelerate cell proliferation, but toxicity has a negative influence. Therefore, high percentages of AP may not be suitable for polymers used as biomaterials. The authors stated that beyond the electroactivity, the introduction of AP changed the irregular structure of the scaffolds to a very regular one, which may be used as a template for the normal differentiation of neuronal or cardiovascular cells (Figure 9).

Researchers have also successfully synthesized novel biodegradable electroactive polyurethanes containing aniline pentamer (AP‐PU) for cardiac tissue engineering uses.^163^ The AP‐PU was blended with PCL at an equal weight ratio to tune the physicochemical properties and biocompatibility. The electrical conductivity of the prepared samples was recorded in the semiconductor range (~10^−5^ S/cm). It has been proved that the conductivity of about 10^−6^ S/cm is sufficient to conduct micro‐current for stimulating neuronal cell proliferation and differentiation since the human body has a lower micro‐current intensity.^252^ However, the semiconductor range of conductivity was still enough.^161^ MTT assays using L929 mouse fibroblast and HUVECs showed that the prepared blend (PB) displayed more cytocompatibility than AP‐PU due to the introduction of a biocompatible PCL moiety. The in vitro cell culture also confirmed that PB was as supportive as the tissue culture plate. However, AP‐PU with the higher AP concentration showed less compatibility than PB containing lower amounts of AP. Therefore, optimization of AP concentration is imperative for acquiring specimens with the most negligible cytotoxicity. The evaluation of the antioxidant activity of conducting polymers and nanomaterials needs to be taken into account when these materials are considered for biomedical applications.^253^ Since conducting polymers are redox‐active, they can be considered reducing agents to scavenge free radicals. 1,1‐diphenyl‐2‐picrylhydrazyl (DPPH) antioxidant assay is one of the most common techniques to study the antioxidant property of materials. The percentage of DPPH scavenging for as‐prepared AP‐PU was recorded at almost 58.9% after 15 min, which may be beneficial for the healing of tissues suffering from high oxidative stress, primarily due to infarction. The results of this study have highlighted the potential application of this electroactive polyurethane as a platform substrate to study the effect of electrical signals on cell activities and to direct desirable cell functions for tissue engineering applications. In another attempt, our group fabricated a scaffold from an aniline pentamer‐modified polyurethane/PCL blend using a mixture of PEG and salt particles in a double porogen particulate leaching and compression molding methodology for cardiac tissue engineering.^104^ The conductivity of the scaffold was measured as 10^−5^ and preserved for at least 100 h post‐fabrication. The electroactive scaffold supported neonatal CMs' adhesion and growth, showing a more extensive effect on the expression of the cardiac genes involved in muscle contraction and relaxation (troponin‐T) and cytoskeleton alignment (actinin‐4) compared with the PCL scaffold as a nonelectroactive substrate and TCP. This result indicated the potential of incorporation of AP as an electroactive moiety for induction of CM proliferation and repair of damaged heart tissue.

Although PANI exhibits proper electrical conductivity, it has poor solubility in common organic solvents. Moreover, it suffers from low processability, nondegradability under physiological conditions, and inflammatory response because of the de‐doping process, limiting its practical use.^238^ Nevertheless, aniline oligomers have attracted gigantic attention because of their proper processability, biocompatibility, degradability, and ease of synthesis. Dopant concentration/type, reaction time, functional groups, and molecular weight of PANI and oligoanilines exhibit essential effects on its biocompatibility and performance.^210^ PANI segments have been utilized to elaborate engineered conductive biocompatible injectable hydrogels for tissue engineering.^217^ It has been demonstrated that oligoaniline segments exhibit different effects on cells due to their various end groups and molecular weights. Carboxylic acid‐end caped aniline pentamer has exhibited better biocompatibility compared to other oligomers.^232^, ^233^ Moreover, it has been confirmed that aniline oligomers enhance cellular adhesion, proliferation, and growth at an optimum concentration. It was explained that the agarose shows low cell adhesiveness and proliferation because of its inert structure.^37^ Grafting aniline pentamer with carboxylic end groups enhances cell adhesion, proliferation, and growth.^138^

Accordingly, the main confusions regarding the application of PANI‐based materials in cardiac tissue engineering can be categorized as cell adhesion problem of untreated PANI, the biocompatibility restriction of PANI from cell to cell, conductivity reduction over time, toxicity problem of PANI when polymerization degree is not controlled, and unpredictable effects of PANI on mechanical behaviors.

Concluding, the literature reports point to the significant limitations on the conductive polymers' application in cardiac tissue engineering, despite their beneficial performance. Regarding the seriousness of the application, materials used as scaffolds should be undoubtedly safe for the human body. Therefore, the application of conductive polymers still requires enormous efforts from researchers to reduce unfavorable effects.

## 5 CARBON‐BASED NANOMATERIALS IN CARDIAC REPAIR

Although using conductive carbon‐based nanomaterials seems a simple way to make the scaffolds conductive, problems associated with size‐dependent,^254^ shape‐dependent,^255^ environment‐dependent,^256^ cell‐dependent,^257^ and/or performance‐dependent^258^ toxicity are still hot challenges. Furthermore, the in vitro and in vivo protein corona effects which can significantly alter the performance and essential characteristics of nanomaterials (e.g., graphene‐based materials) at the personalized‐dependent level are still a very important issue.^259^ In this regard, more detailed research on conductive nanomaterials has been performed with high attention over the last two decades. Carbon‐based materials (CBMs) have gained significant attention in the path of regenerative medicine over the past few decades. Particular features and characteristics of these nano‐sized materials, ranging from tens to hundreds of nanometers,^260^ are the reason for the amount of attention turned toward them. Herein, an introduction to CBMs is presented, followed by a literature review of the most recent applications of frequently used CBMs in cardiac tissue engineering.

**FIGURE 10:**
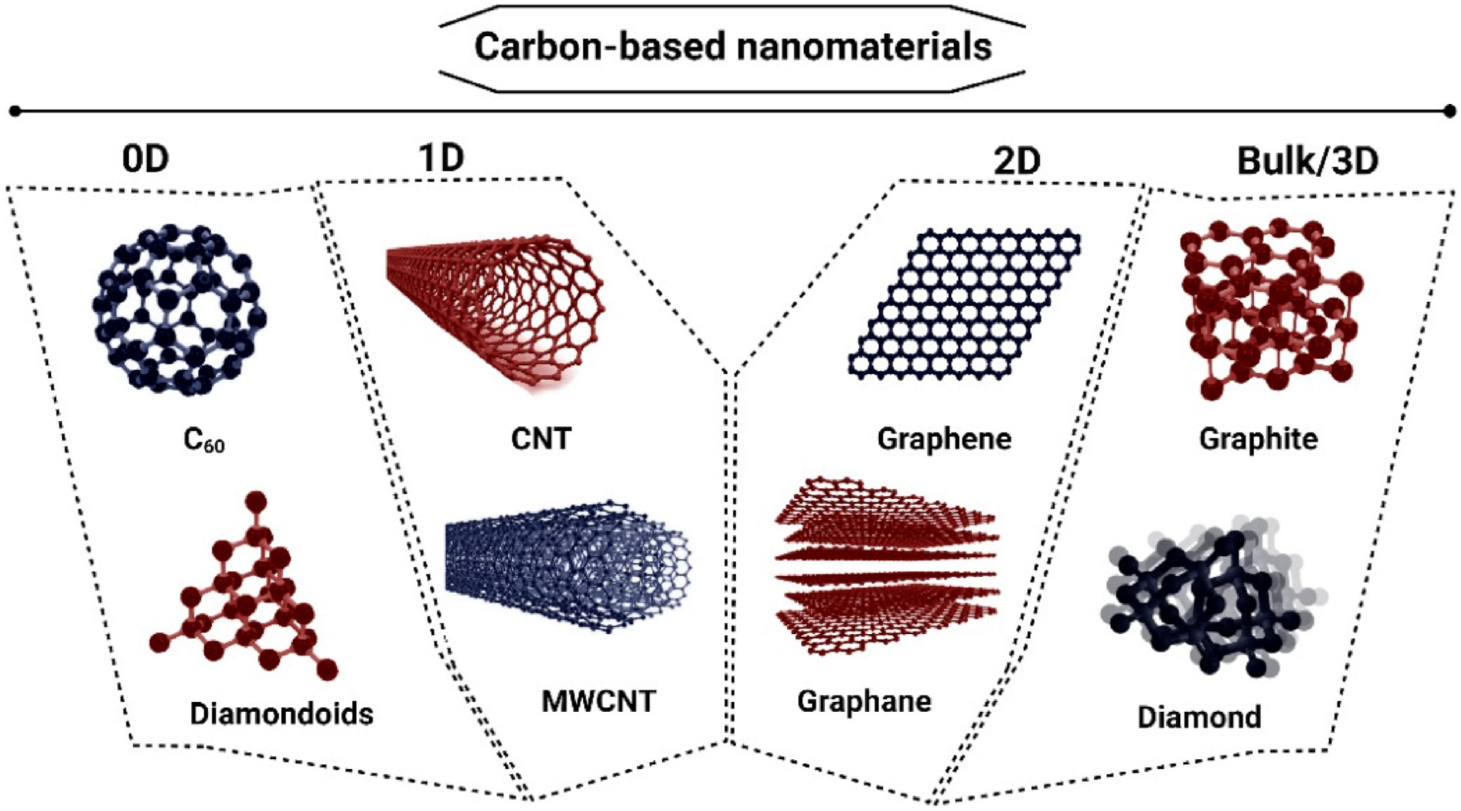
Schematic from chemical structures of some carbon‐based materials categorized by dimensionality

Figure 10 represents some of the most famous carbon‐based nanomaterials' structures. Sheets of *sp* ^2^‐hybridized carbon atoms composed of three balanced orbitals are the primary constituent units in graphene‐based materials (GBMs).^261^ Illustrating their structure, an *s* orbital and two *p* orbitals equal in energy level, positioned at 120°angles, form strong σ‐bonds. Thereby, a trigonal planar geometry exhibits within the layers of GBMs. High mechanical stiffness and extreme chemical and thermal stability are represented, owing to the strong σ‐bonds. Apart from that, the potency of easy electron excitement from the valence into conduction bands gives rise to the remarkable thermal and electrical conductivity of CBMs.^261^ Networks of *sp* ^2^‐hybridized carbon atoms do facilitate the conduction of electrons to move within the lattice and transfer charge freely. The electrical conductivity of GBMs in parallel to the mentioned features is highly favorable since, as already explained, the electrical coupling of the constructed substrates and cells is of tremendous importance in tissue engineering, cardiac especially. Therefore, GBMs alone or in combination with other materials provide the opportunity of optimized and enhanced electrical, physicochemical, and mechanical properties.

Single or multiple layers of the described structure produce different allotropes of GBMs. Graphite is one common allotrope of amorphous solid carbon in which hexagonal networks of *sp* ^2^ hybrid orbitals form stacking layers.^262^ The remaining orbital in this structure, positioned perpendicularly, forms π‐bonds between two adjacent layers, creating a three‐dimensional structure. The relatively weak bonding gives rise to Van der Waals bindings,^261^ providing easy layers' separation potential. Graphene, two‐dimensional single layers of *sp* ^2^‐hybridized carbon atoms, is isolated from graphite through various methods, including physicochemical/mechanical exfoliation of graphite (such as exfoliation by thermal shocking^263^ and ion intercalation^264^), epitaxial growth, and chemical vapor deposition.^265^, ^266^ Up till 2004, before the very first‐time graphene was successfully isolated, the idea of single‐atom‐thick materials under ambient conditions was considered thermodynamically unstable.^267^, ^268^

Another significant characteristic of these materials is the large surface area per unit, leading to their specific behaviors.^269^, ^270^, ^271^ Producing graphene oxide (GO), a derivative provided via different levels of oxidated graphite, can be performed with various methods, among which Hummers is by far the most common method. Hummers is a less time‐consuming method and has the advantage of excluding ClO_2_ gas production.^267^ Oxygen‐containing functional groups within GO, including carboxyl, carbonyl, hydroxyl, and epoxide groups,^272^ bring about the superiority of hydrophilicity, solubility in aqueous environments,^273^ real blood samples^264^ and in vivo applications,^274^ the capability of drugs^275^ and nanobubbles^276^ loading, and easy bio‐functionalization^277^ compared to pure graphite or graphene, which makes the GO materials highly desirable in biological applications required further supports of cell adhesion.^278^ Depending on the synthesis method, the carbon to oxygen ratio of GO varies from 1.8 to 2.3.^279^ The presence of functional groups causes an absence of the electron‐spreading pathways in carbon networks. In further detail, saturated *sp* ^3^ carbon atoms exist within GO, bound into the oxygen‐containing chemical groups. This creates energy gaps between the valence and conduction electrons emerging insulating properties in GO,^188^ which may be counted as a disadvantage in the applications that required a metallic behavior.^280^ Resistivity varies as a function of the C/O ratio. The more the oxidation level gets, the more carbon atoms saturate, thus fewer pathways facilitate charge distribution, and resistivity appears.^279^ However, depending on the oxidation level, incorporating GO sheets into resistive materials may provide different conductivity levels, as reported in many studies.

Shortages and deficits of GO are somewhat overcome by partially reducing via a reduction treatment.^281^ Reduced graphene oxide is an optimized version recovering favorable properties of both graphene and graphene oxide.^281^ As a result, better biological behavior is achieved in addition to electrical conductivity, which is desirable in any tissue engineering. The raised concentration of ROS species due to the body's inflammatory response causes substantial incompetent adhesion of cells within the infarcted area when it comes to MI. Thereby, the incorporation of hydrophilic GBMs in cardiac tissue regeneration approaches is desirable. Great binding sites or functional groups within the structure give rise to enhanced adhesion of cells to GBM‐containing substrates.^282^

Carbon nanotubes (CNTs) are graphene sheets rolled up in the shape of one‐dimensional hollow cylinders, with diameters of few nanometers and a height of up to several micrometers. Substantial high aspect ratios are obtained as a result. Single‐walled carbon nanotubes (SWCNTs) with diameters of 0.8–2 nm^273^ are made of a single sheet, while a number of 2–50 sheets together make up multi‐walled carbon nanotubes (MWCNTs) possessing a diameter of 5 nm on average.^273^, ^283^ CNTs are higher contaminated compared to graphene, especially due to their catalysts contained. Therefore, along with its water insolubility, uncertain biocompatibility is a topic of much debate. Hence, time‐consuming purification processes (such as plasma etching) are required in the case of biological applications.^284^

Bent graphene sheets creating concentric (coaxial) nanocones in rows with diameters in the range of 50–500 nm are called carbon nanofibers (CNFs).^285^ Both CNTs and CNFs are ideal candidates to be exploited in composites as reinforcing particles. However, in comparison, more defects are presented within the structure of CNFs. Less conductivity and weakened mechanical properties, as a result, are observed.^129^, ^286^, ^287^ Moreover, CNFs are acknowledged to be more toxic than CNTs, none the less it strongly depends on the synthesis and processing method.^288^

Zero‐dimensional spherical structures called fullerene, composed of diverse numbers of hexagonal rings and a constant total of 12 pentagonal rings of carbon atoms,^289^ are also widely investigated. Due to the presence of pentagons, hence a curved not planar network of carbon atoms, hybridizations between *sp* ^2^ and *sp* ^3^ are presented within the structure.^290^ The superior biocompatibility of fullerene compared with graphite and CNTs, along with other particular features, makes them favorable.^291^

A distinct character of zero‐dimensional fullerene and its derivatives is the ability to penetrate cell membranes owing to considerably small diameters of about 0.7 nm, the smallest among all the CBMs allotropes. Therefore, appealing manipulations of cell behavior seem to be possible through the use of fullerene nanoparticles. Substantially small size and spherical shape make fullerene feasible as radical scavengers and antioxidants.^292^, ^293^ However, the hydrophobic nature of fullerene brings up attention to its derivatives.^290^ Fullerenol with higher water solubility is satisfyingly recovering.^294^

Carbon dots (CDs) or carbon quantum dots are also zero‐dimensional, either crystalline or amorphous materials, consisting of primarily *sp* ^3^‐hybridized carbons as well as *sp* ^2^ orbitals. They typically possess diameters in the range of 2–10 nm.^290^ Specific optical properties due to the broadband of wavelength absorption (260–320 nm) give rise to the photoluminescence radiation feature in CDs, which in addition to electrical conductivity, low cytotoxicity, and water solubility, is a promising feature for biological applications.^273^, ^290^, ^295^

Diamond, another amorphous form of carbon, is majorly known for its remarkable hardness. Diamond exhibits insulating properties as it is composed of saturated *sp* ^3^‐hybridized carbon atoms in a tetrahedral geometry. Carbon nanodiamonds (CNDs), spherical particles possessing a diameter of about 5 nm,^290^ have also gained attention because of their prosperous features. Relatively small diameters, large surface‐to‐volume ratio, and specific optical properties are provided.^273^ Semiconductor quantum dots of CNDs are assumed to be the least toxic among all carbon allotropes discussed above.^204^, ^296^

CBMs, particularly GBMs, mostly graphene and CNTs, have been widely used in cardiac tissue engineering due to their specific properties already mentioned. Diverse mechanisms of promoting cell differentiation are the reasons for the amount of attention turned toward them, owing to their unique physical, chemical, and mechanical properties. These promoting mechanisms typically originate from features like the potency of mechanical supporting, stability in aqueous environments, the opportunity to be functionalized, large surface area, topography and presence of nanoroughnesses, and finally, yet notably, their electrical properties.^297^ Herein, among all the desirable features of CBMs and different mechanisms affecting cellular interaction of CBMs, the influence of electroactivity has been reviewed in particular.

### 5.1 Graphene application in cardiac repair

**FIGURE 11:**
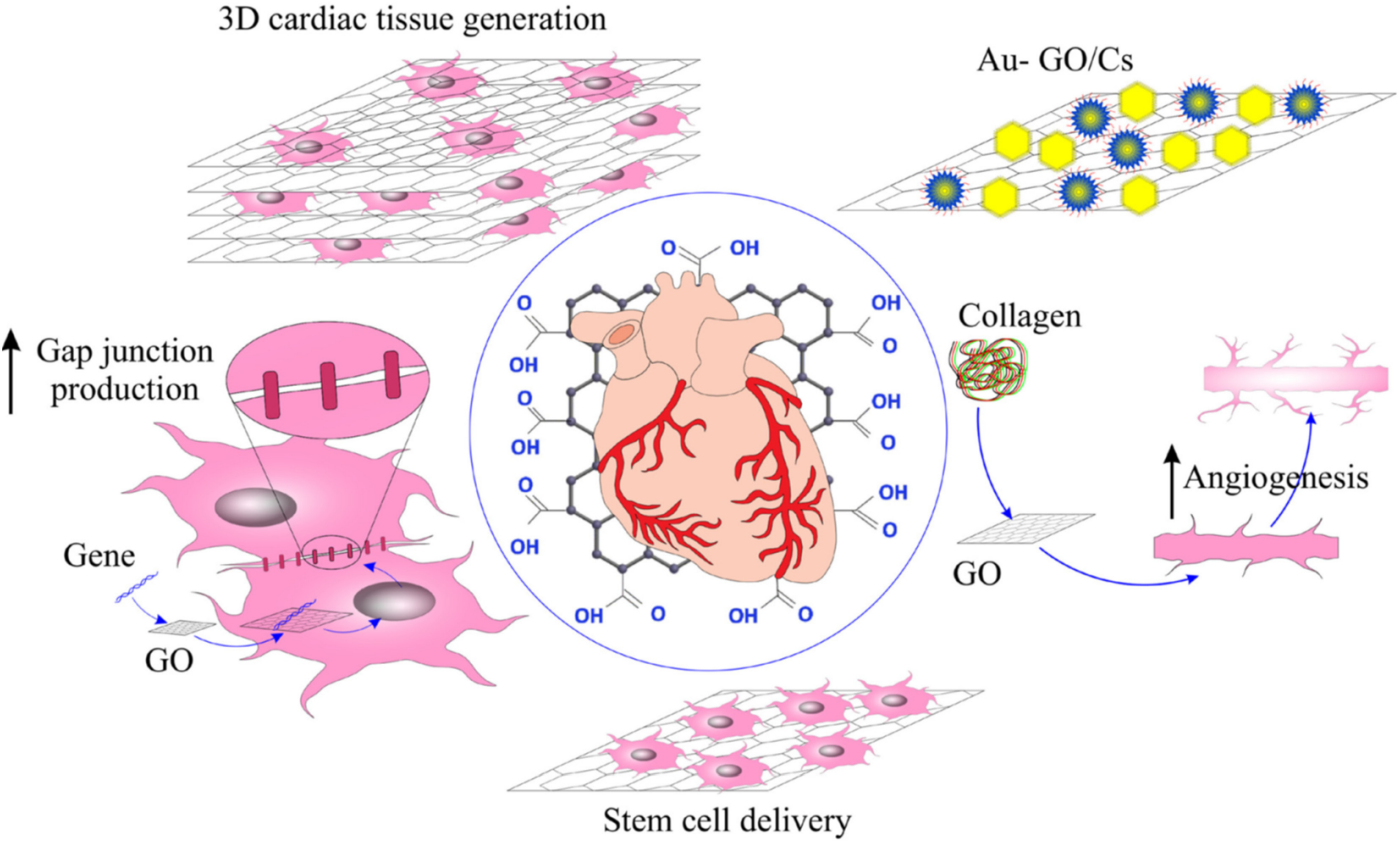
A schematic diagram of multiple applications of graphene oxide in cardiac regeneration^299^

The first studies employing graphene nanosheets approaching cardiac tissue regeneration were conducted in the early 2010s. In a study conducted by Kim et al.,^298^ graphene sheets were assessed for biocompatibility evaluations with primary adult CMs for the first time. Adult rat ventricular myocytes were cultured on graphene‐coated coverslips, and cell viability, contractility, and intracellular calcium ion dynamics were evaluated. The obtained results were perfectly supportive for graphene nanoparticles along with cardiac cells. Graphene substrates did not disturb cells viability and significantly enhanced cell attachment as regards control. Properly functional CMs, similar calcium transient activity, and sensitivity in the graphene company were also observed. The studies showed that a synthetic multipart such as the macrophage‐targeting/polarizing GO complex was capable to reduce ROS from macrophages. In a study, a mixture of macrophage‐GO complex and IL‐4 pDNA was applied to induce differentiation of M1 to M2 macrophages and secretion of regenerative cytokines for cardiac. As a result, it reduces inflammation as well as increases differentiation into M2 macrophages and improves heart function in animal models (Figure 11).^299^

Park et al.^300^ also evaluated the potency of graphene sheets for cardiomyogenic differentiation of mesenchymal stem cells (MSCs). Biocompatibility was first approved, then examined cardiomyogenic markers at the transcript level in graphene substrates lacking any exogenous chemical inducers. Several markers, including cardiomyogenic transcriptional factors, cardiomyogenic contractile proteins, and gap junction proteins, were assessed for expression, and enhanced levels were reported. Expression of genes making appropriated ECM proteins and related cell signaling molecules were also observed to be enhanced. Nevertheless, the enhanced electrical conductivity of the substrates within double‐layer and triple‐layer graphene was not likely to contribute to the differentiation as no significant difference was evidenced. As a result, particular ECM proteins and cell signaling molecules' upregulated gene expression were only suggested to be responsible.

Another pioneering study to mention is the one carried out later by Lee et al.^301^ Interestingly, undifferentiated human embryonic stem cells (hESC) were cultured with graphene‐coated coverslips about Matrigel‐coated glass and glass coverslips. The aim was to observe whether the cardiomyogenic differentiation of cells in the presence of graphene was enhanced. Significantly higher levels of cardiomyogenic transcriptional factors, cardiomyogenic contractile proteins, and gap junction proteins have been reported. However, this special promotion is only attributed to the specific physical characteristics and topography of the graphene particles, and the influential character of the enhanced electrical conductivity was not stated.

Ahadian et al.^302^ later embedded graphene nanoparticles into mouse embryoid bodies (EBs) compared with graphene‐free EBs characteristics. As a verification of the higher conductivity of the EB‐graphene sample, higher electrical current at a constant voltage and fewer impedance values were observed. Electrical impulses as stimulation were also applied on day 4 of culture for 2 consecutive days. Results showed an upregulation in cardiomyogenic contractile factors of cardiac protein troponin T (cTnT) and cardiac muscle troponin T (Tnnt2) expression due to graphene presence and electrical stimulation. Notably, stimulated graphene‐free EBs showed a higher cTnT expression than nonstimulated graphene‐containing EBs, highlighting the importance of electrical stimulation and conductivity as a means to investigate cardiomyogenesis. More differentiated CMs and blood cells were observed on graphene‐containing EBs, and characterized beating activity was significantly enhanced on stimulated graphene‐containing EBs compared to other experimental groups.

Furthermore, Ameri et al.^303^ created a 3D graphene foam through the chemical vapor deposition method, in which they seeded HL‐1 cells derived from mouse atrial tumors. Rapid cell adhesion to the scaffold and encouraging contractile behavior of cells within 3 days after seeding continued up to 9 days on graphene compared to the culture flask (5 days) was observed.

Later on, a novel electroactive PEG‐based graphene‐containing scaffold was fabricated in which anisotropic electrical conductivity was featured.^100^ Regarding the natural topography of the heart muscle, anisotropic topographic constructs are more likely to mimic properly. Cell morphology, protein expression, calcium ion transient, and action potential evaluations did approve the beneficial effects of graphene, yet better results were obtained when an oxygen‐plasma exposure was applied on the surface of the scaffolds. Anisotropic electrical conductivity, significantly lower electrical resistivity, the fabricated structure, and improved hydrophilicity of treated surfaces are the leading causes.

In another study, Wang et al.^304^ prepared a 2D monolayer sheet of graphene (via the chemical vapor deposition technique) in which electrical transfer characteristics showed excellent electron mobility and conductivity. Contracting CMs were identified within 7–9 days after hiPSCs seeding, even in the absence of electrical stimulation. Coverslips were employed as control groups as immunoassay results showed a higher cTnT and α‐actinin, cardiac contractile protein expression on graphene sheets. Quantitative RT‐PCR results represented significantly increased levels of MEF2c, GATA4, and NKX2‐5, genes known to play roles in cardiac development. Additionally, cardiac genes of ACTC1, TNNT2, and Cx43 also showed elevated levels of expression. The amount of Cx43 was two‐fold higher in graphene sheets compared to coverslips through Western Blot analysis. Besides, RyR and SERCA2a gene expressions as an assessment of calcium ion kinetics were also determined, showing higher levels in graphene‐based hiPSC‐derived CMs. Bone morphogenetic proteins (BMP) signaling to promote cardiogenesis was also evaluated, and confirming results were further observed. Notably, oxygenated modified graphene was obtained to cause a significant decrease in BMP4, MEF2c, and GATA4 expression levels due to reduced conductivity due to oxygen's addition. Providing electrical signal propagation, the constructed graphene sheet, in brief, was likely to mimic the native myocardium and promote hiPSC‐derived CMs' maturation properly.

Bahrami et al.^91^ fabricated 3D and 2D conductive graphene foam scaffolds and reported an over‐expression of Conx43 and TrpT‐2 in seeded cells in both experimental groups compared to tissue culture plates as control. 3D graphene foam with a reported conductivity value of 9 Scm^−1^ notably showed the highest levels of Conx43 expression holding and confirming the great promise over conductivity as well as the porous structure of graphene foam substrate.

**FIGURE 12:**
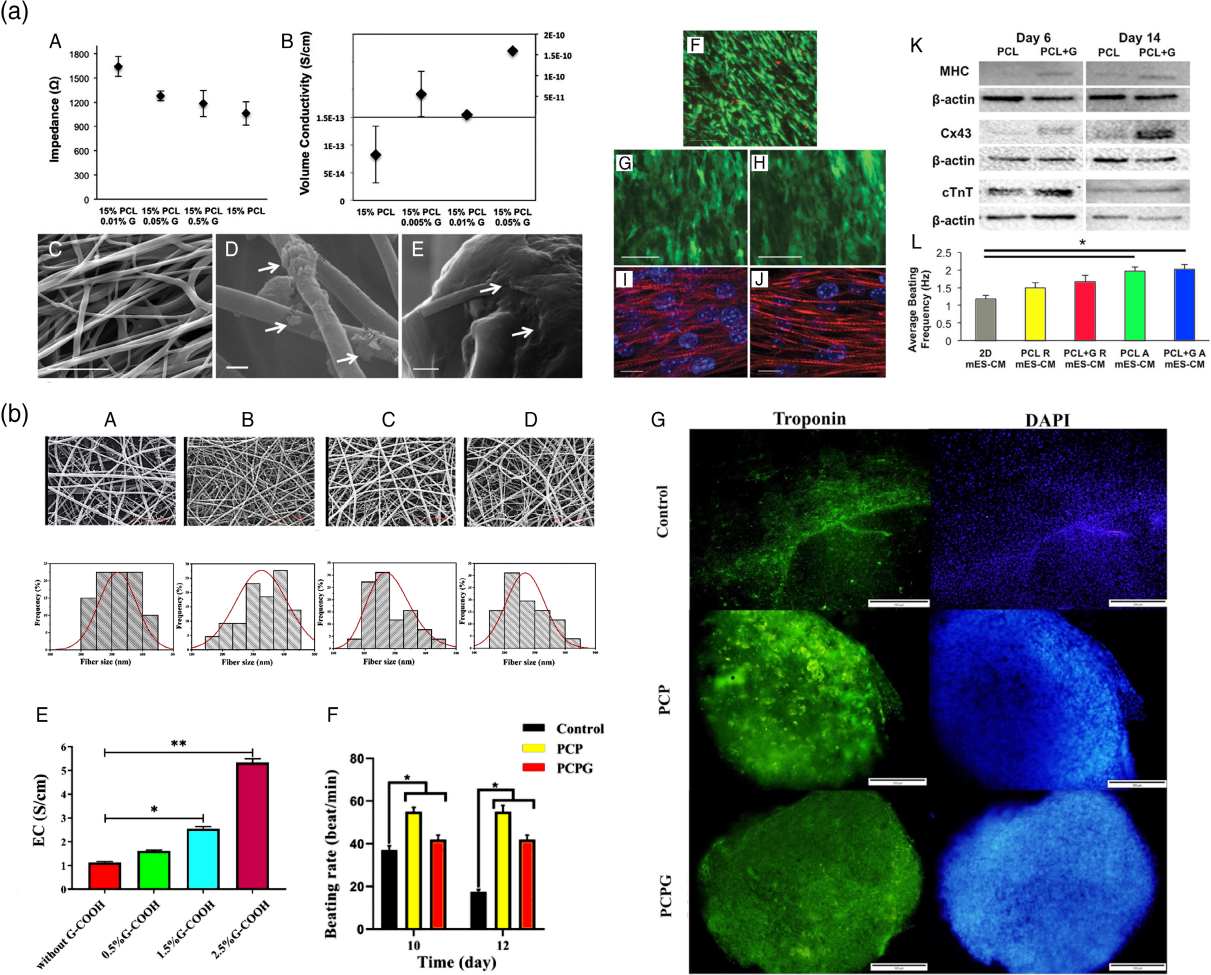
Graphene application in cardiac tissue repair. (a) Electroconductive three‐dimensional (3D) nanofibrous graphene and poly(caprolactone) (PCL + G) composite scaffold for cardiac tissue engineering. (A) Impedance analysis of 15% PCL and 15% PCL + G scaffolds with various concentrations of graphene indicated a decline in impedance with higher graphene concentrations. (B) Volume conductivity of random PCL scaffolds with various graphene concentrations showed higher conductivity with an increase in graphene concentration. (C) SEM image of aligned 15% PCL + G (0.01%) scaffold. (D) Graphene particles within the matrix showed by arrows. (E) Graphene particles edge on a fiber shown by arrows. (F) Live/Dead analysis indicating high cell viability on PCL + G scaffolds on day 7. (G, H) High expression of cTnT‐eGFP by cells cultured on PCL or PCL + G scaffolds on day 6, respectively. (I, J) well‐registered sarcomeres aligned along the major fiber axis of PCL and PCL + G scaffolds, respectively. (K) Increased expression of MHC, Cx43, and cTnT was indicated in mouse embryonic stem cell‐derived cardiomyocytes (MES‐CM) cultured on scaffolds. (L) Average beating frequency of MES‐CM on day 6. Reproduced from Reference 305 with permission from Wiley. (a) Electroconductive graphene‐containing scaffolds composed of PCL, chitosan, and polypyrrole for cardiac tissue repair. (A–D) Representative SEM images of PCL/chitosan/PPy blend films with various concentrations including PCPG0, PCPG0.5, PCPG1.5, and PCPG2.5, respectively. (E) Representative TEM image of fibers containing graphene. (F) Beating rate (BPM), and (G) immunofluorescence identification of embryonic bodies by troponin (green) and counterstained by DAPI (blue). Reproduced from Reference 306 with permission from the American Chemical Society

In another study conducted by Hitscherich and company,^305^ graphene was used within a PCL substrate to prepare nanofibrous composite scaffolds for cardiac tissue engineering. As higher graphene concentration decreased electrical impedance, local conductive sites were confirmed to exist. These sites were likely to facilitate point electrical stimulation propagation, thus offering promising potential as an excellent approach for cardiac tissue engineering. Upregulation of cardiac‐specific markers including cTnT, Cx43, and myosin heavy chain (MHC) was observed in all experimental scaffolds, among which aligned scaffolds containing graphene showed the best results, compared to randomly oriented and graphene‐free ones. Calcium ion handling properties were also significantly improved, and favorable contracting properties were observed (Figure 12).

In another recent study, Talebi et al.^307^ created graphene‐containing scaffolds composed of PCL, chitosan, and polypyrrole. Embryonic stem cells formed as EBs were cultured on graphene‐containing and graphene‐free scaffolds and gelatin‐coated tissue culture plates as reference groups. Raised level of Troponin expression and improved beating rate was observed in graphene‐containing scaffolds compared to other experimental groups, making them potentially applicable for cardiac regeneration (Figure 12).

### 5.2 Graphene oxide application in cardiac repair

As mentioned above, graphene oxide should be considered an auspicious component for the materials developed for CVDs treatment. Table 3 summarizes the most promising literature reports dealing with GO‐containing biomaterials applied in cardiac tissue engineering.

**TABLE 3:** The GO‐containing biomaterials USED in cardiac tissue engineering

Conductive substrate	Properties	Conductivity or resistance	Biological effect	References
Graphene oxide/chitosan	Porous structure	0.134 S m^−1^	Good cell viability, promotion of cell attachment and intercellular network formation, and upregulation of the cardiac‐specific gene and protein expression involved in muscle conduction of electrical signals (Connexin‐43)	308
Graphene oxide/collagen	Randomly oriented interconnected pores, 162 kPa tensile strength	~10^−4^ S m^−1^	Supported neonatal CMs' adhesion and upregulated the expression of the cardiac genes, including Cx43, Actin4, and Trpt‐2	188
Polyethylene terephthalate/graphene oxide	Electrospun core–shell nanofibers, a diameter of 253 ± 67 nm	1.3 × 10^−6^ S cm^−1^	Supports human umbilical vein endothelial cells' spreading morphology and CM elongated morphology	309
Hastalex (functionalised graphene oxide and poly[carbonate‐urea]urethane)	Contact angles of Hastalex surfaces (85.2 ± 1.1°), tensile strength 57.1 MPa	N/A	No negative effect on the RBC membranes, a moderate macrophage infiltration had been detected	310
Reduced graphene oxide foam templated by nickel foam	organ‐on‐a‐chip engineering of cardiac constructs	1.12 S cm^−1^	Good cell adherence, spreading, activity, organization, and beating function	158
oligo(poly(ethylene glycol) fumarate)/graphene oxide	Hydrogel, injectable	4.235 × 10^−3^ S cm^−1^	Improve cell attachment, enhanced the Ca^2+^ signal conduction of CM in the infarcted region, enhanced the generation of cytoskeletal structure and intercalated disc assembly	311

Probing the impacts of GO incorporation in the path of cardiac repair, in original research by Shin et al.,^312^ the hybrid hydrogel of gelatin methacrylate (GelMA) containing GO sheets compared to the new GelMA hydrogels were studied. The improved viability confirmed beneficial impacts and nontoxicity of GO sheets within the prepared hydrogels, adhesion, spreading, and proliferation of cultured cells on Go‐GelMA hydrogels compared to control. Decreased electrical impedance and increased electrical conductivity were observed in GO‐GelMA hydrogels, ascribed to reducing amine groups in the GelMA structure. This feature is favorably applicable in cardiac regeneration induction as a function of GelMA methacrylation. The amount of amine functional groups can adjust reduction levels on GO, thus electrical conductivity values.

Moreover, the GO‐GelMA hydrogel was used in another study^313^ as a substrate for cell seeding. Additionally, poly‐l‐lysine (PLL)‐coated GO sheets were applied to them before adding the next same layer of GO‐GelMA. PLL‐coated GO layers were likely to facilitate layers' adhesion and induce electrostatic attractions. PLL is later enzymatically degraded, resulting in the recovery of GO particles' electrical conductivity. Therefore, the use of PLL‐coated GO sheets in between layers of cell‐seeded hydrogel provides the potential of optimizing electrical conductivity. Enhanced cell–cell interactions and supported action potential propagation were attained. Mentioned features and mechanical properties leading to more integrity altogether brought about a proper electrical coupling and promoted maturation of the seeded cells.

**FIGURE 13:**
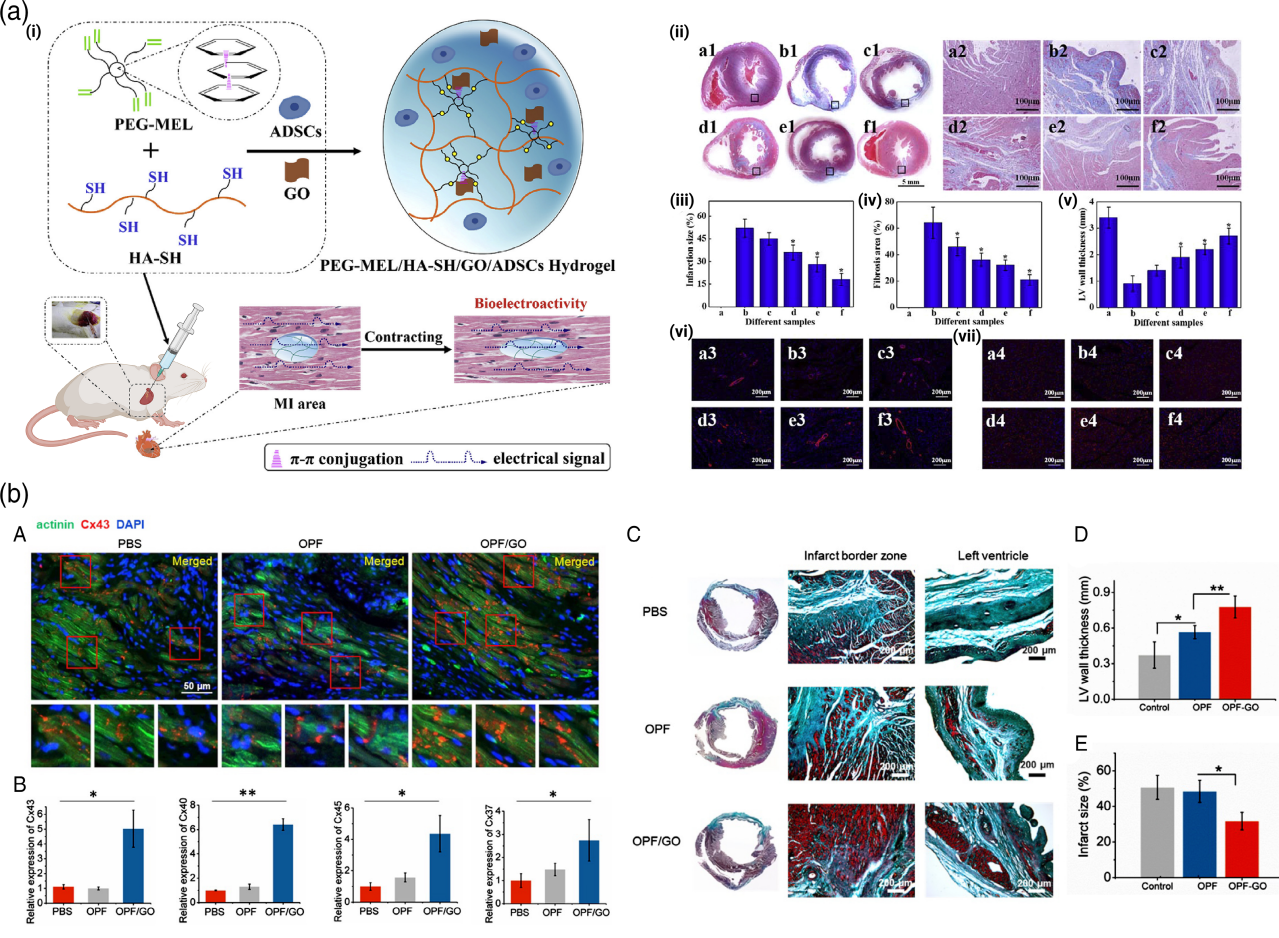
Graphene oxide application in cardiac tissue repair. (a) An injectable conductive hydrogel utilizing π‐π conjugation for cardiac tissue repair. (i) Schematic illustration of the application of soft and conductive PEG‐MEL/HA‐SH/GO hydrogel system for cardiac tissue repair. (A, A1–A4: Sham; B, B1–B4: PBS; C, C1–C4: PEG‐MEL/HA‐SH; D, D1–D4: PEG‐MEL/HA‐SH/GO; E, E1–E4: PEG‐MEL/HA‐SH/ADSCs; F, F1–F4: PEG‐MEL/HA‐SH/GO/ADSCs). (ii) Masson's trichrome staining for collagen (blue) and muscle (red); A2–F2 is a magnification of the corresponding black box labeled in A1–F1. (iii) Infarction size. (iv) Fibrosis area. (v) LV wall thickness. (vi) Immunofluorescence staining for a‐SMA. (vii) Immunofluorescence staining for Cx43. * shows a significant difference between the experimental group and PBS treated group. Reproduced from Reference 155 with permission from Elsevier. (b) Injectable oligo(poly(ethylene glycol) fumarate) (OPF)/graphene oxide hydrogels for cardiac tissue repair. (A) Immunofluorescence staining indicated increased gap junction remodeling in the infarcted region of the OPF/GO hydrogel‐treated group compared to the OPF‐ and PBS‐treated groups. (B) qPCR analysis of the expression levels of gap junction‐associated markers Cx43, Cx40, Cx37, and Cx45. (C) Masson trichrome staining of PBS‐, OPF‐, or OPF/GO‐injected heart 4 weeks after injection. (D) Left ventricle wall thickness and (E) infarct size measured for weeks post‐injection. Reproduced from Reference 311 under the terms of CC‐BY license open access

Contrastively, a soft injectable hydrogel utilizing π–π conjugation in which long‐range electron conduction is facile was fabricated by Bao and colleagues.^155^ Relative softness was achieved by emanating multi‐armed polyethylene glycol diacrylate (PEGDA) from the melamine core with an π–π conjugation ring. GO incorporation aiming promoted mechanical and electrical properties were followed. Adipose‐derived stem cells (ADSCs) were then encapsulated, and an in vivo experimentation aiming for cardiac repair was conducted on Sprague–Dawley (SD) rats. Other than the conductivity resulting from the presence of π–π conjugation, hydrogels electroactivity further increased after loading GO and reached an appropriate amount of 2.84 × 10^−4^ S cm^−1^, which is right within the range of that in native myocardium. Overall, the best results were associated with the GO‐containing group of experiments in which the most elevated Ejection Fraction and Fractional Shortening were observed. Meanwhile, the highest recovery of infarction size and fibrosis area, more LV wall thickening and muscle‐like tissue forming, and maximum Cx43 expression and neovascularization were all attributed to the mentioned experimental group. Upregulated expression of α‐SMA as well resulted in promoting systolic and diastolic function of rats in infarcted hearts treated with GO‐containing hydrogels (Figure 13a).

Zhou et al.^311^ also obtained an injectable semiconductive GO‐incorporating hydrogel based on oligo (poly (ethylene glycol) fumarate) (OPF). As predicted, OPF/GO hydrogels showed better conductivity as they could readily facilitate muscle contraction compared to the pristine OPF. As a result of better mechanical and electrical features of OPF/GO hydrogel, both qPCR and immunohistochemistry analysis indicated substantially higher degrees of gap junction proteins expression (Cx43, Cx40, Cx45, and Cx37). Western blotting also confirmed GO contribution superiorities. Phosphorylation levels of GSK‐3β hence, β‐catenin signaling, were improved, which then promoted CX43 expression. Angiogenesis was also demonstrated, which was suggested to be resulting from gap junction proteins overexpression. Moreover, highly restored cardiac function due to LV wall thickening and reduction in infarct size was observed in OPG/GO injected rats. Improved EF and FS were confirmed via echocardiography 4 weeks post‐MI. Ventricular performance as well as significantly enhanced. Additionally, CMs isolated from OPF/GO injected hearts did promote calcium transient signal conduction. This was attained by determining calcium ion fluctuations of isolated CMs 4 weeks after MI (Figure 13b).

Jing et al.^314^ later designed and synthesized another injectable and conductive hydrogel composed of chitosan and graphene oxide with innovative features of self‐adhesiveness and self‐healing. Polydopamine (PDA) was employed, produced by the oxidative polymerization of dopamine through which graphene oxide's partial reduction occurred from about 54.2% to 32%. This resulted in electrical conductivity of 1.22 × 10^−3^ S cm^−1^ in the GO‐containing experimental group. However, an optimized value of GO concentration was determined as excess amounts lowered conductivity. A mildly decrease in electrical conductivity at concentrations over a specific value was attributed to the appearance of aggregated GO sheets disrupting charge transmittance. At this optimized value of GO concentration, not surprisingly, conductive GO‐containing hydrogel showed better biological features, including cell viability, proliferation, and beating behaviors compared to other experimental groups, including TCP, GO‐free hydrogel, and other GO concentrations.

Further studies were performed on GO‐containing scaffolds or injectable gels regarding their electrical properties on cardiac tissue engineering, showing the same promising results. Zhao et al.^315^ fabricated a novel injectable Reverse Thermal Gel which transitions from a sol to a gel phase. A conducting resistance of 144.3 kΩ ± 4.3 is reported for RTG‐GO samples which is significantly lower than the control group, thus exhibiting more conductivity.

Conductive scaffolds composed of chitosan and GO were further studied by Jiang and colleagues.^308^ Electrical conductivity measurement was taken and is reported to be 1.34 × 10^−3^ S cm^−1^ which is likely to be favorable in cardiac tissue engineering regarding the native myocardium conductivity as already mentioned (part 4).

Later, electroactive substrates such as cardiac patches composed of polyethylene terephthalate (PET) and GO were fabricated by Ghasemi et al.^309^ using the electrospinning method. Conductivity was measured before and after GO addition, both in solid or core–shell nanofibrous structure—a shell of GO solution on the core of PET fibers. Reported values of 1.2 × 10^−2^ and 1.3 × 10^−2^ S cm^−1^ indicate a higher conductivity on both GO‐containing samples than on control. Promoted cell interactions were demonstrated as a consequence of GO addition.

**FIGURE 14:**
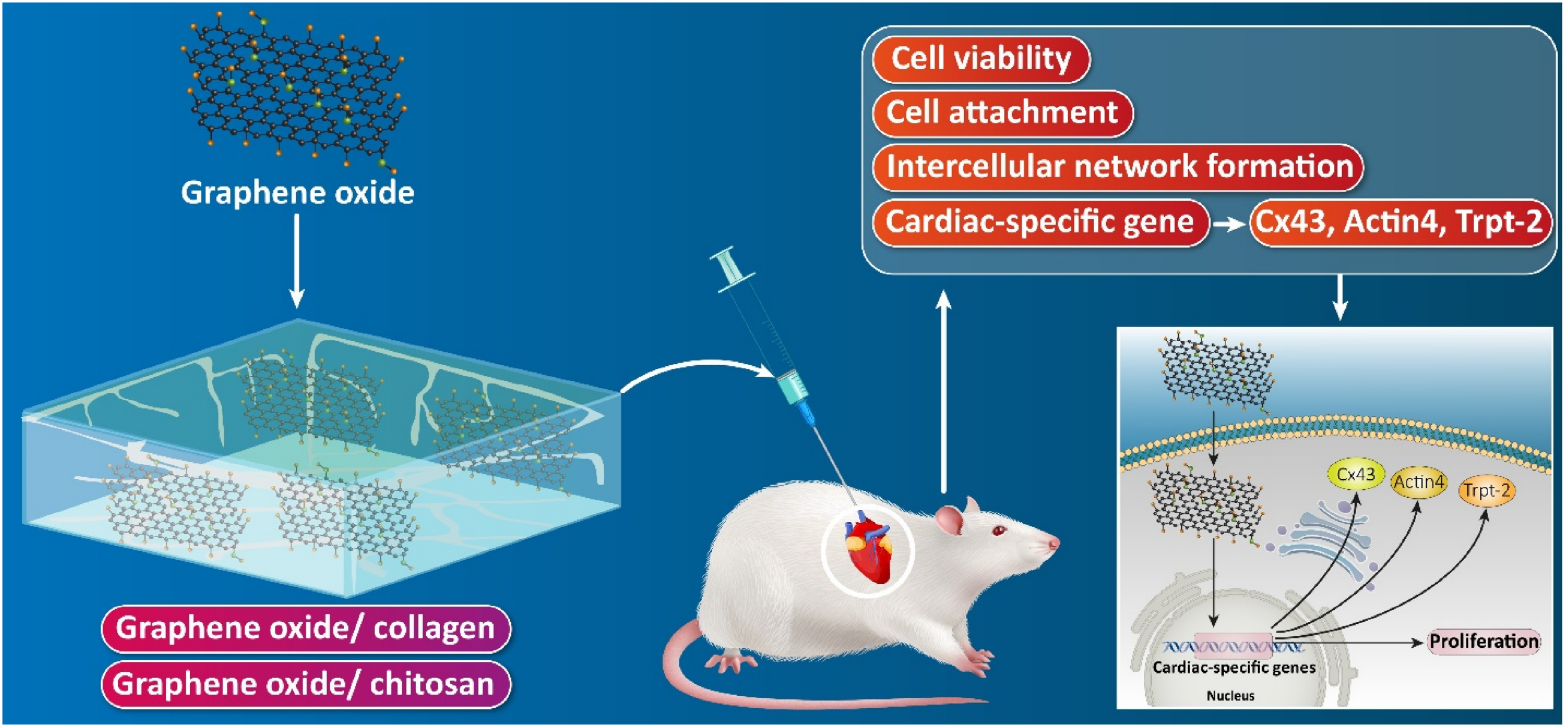
The application of graphene oxide in cardiac repair

Another newly conducted research by Sekula‐Strjewska et al.^278^ examined the influence of different sizes, reduction levels, and layer thicknesses of GO flakes on the culture of human umbilical cord mesenchymal stem cells (hUC‐MSCs). Graphene‐based substrates were fabricated to determine their cardiac and angiogenic differentiation potency of hUC‐MSCs. While a value of 0.04 Ω·cm was calculated for the highly reduced GO (conducted by sodium hypophosphate), lowly reduced GO (conducted by l‐ascorbic acid) exhibited an electrical resistance of 0.85 Ω·cm. This confirms the influence of different levels of reduction on the presence of oxygen atoms, less resistance, and higher electrical conductivity as a consequence. Cells in two different experimental groups were cultured on plates coated with 10–20 μm sized GO‐containing solutions (GO‐lf) and partially reduced with polyphenon60 GO‐containing solutions (rGO‐lr‐P60). Stimulated angiogenic differentiation was observed in both groups; however, rGO‐lr‐P60 cells necessarily required a differentiated culture medium as external stimulation. Significantly improved GATA‐2, ENDOGLIN, and VE‐CADHERIN expression levels were noticed as well as capillary bed formation. Longer‐length capillaries were again observed in the first group of experiments. Moreover, both substrates did support and significantly enhance cardiomyogenic differentiation through the induced expression of factors such as GATA‐4, actin alpha cardiac muscle 1(ACTC1), and myocyte‐specific enhancer factor 2C (MEF2C). Notably, no necessity for special differentiated stimulating mediums is observed (Figure 14).

### 5.3 Reduced graphene oxide application in cardiac repair

Different levels of reduced graphene oxide have also captured the tremendous interest of researchers in the various fields of nanomedicine, including cardiac regeneration. Beginning with some of the elementary studies using rGO, rGO incorporated MSC spheroids (Sph‐rGO) were evaluated by Park et al.^316^ for myocardial repair. Significantly increased expression of Cx43, whether both in vitro and in vivo, was noticed in Sph‐rGO compared to the same size GO flakes‐containing spheroids. This is attributed to the higher conductivity of rGO flakes. The cardiac repair was also evaluated by in vivo injections of Sph‐rGO and rGO‐free MSC spheroids and rGO flakes alone and PBS as a control to infarcted hearts of mice. Cardiac function parameters were assessed, including left ventricular internal diameter at end‐diastole and systole, ejection fraction, and fractional shortening. Each was remarkably improved in (Sph‐rGO) group, attributed to the angiogenic stimulations in MSCs. A reduced area of fibrous tissue was also observed in Sph‐rGO injected myocardium.

Shin et al.^189^ later fabricated and characterized rGO‐containing GelMA hydrogels. Electroconductivity was approved in fabricated hydrogels through the calculation and comparison of electrical impedance values. RGO flakes helped protein adsorption; hence enhanced cell adhesion was observed in rGO‐GelMA hydrogels compared to control. Cardiac tissue culture on rGO‐GelMA exhibited a generally aligned and electrically coupled structure, thus showing better contractile properties. A significantly higher rate of spontaneous beating was also obtained. Properly formed cell junctions, as well as the intrinsic conductivity of the hydrogels, are likely to be contributing. Extrinsic electrical stimulations at different frequencies were also employed, and appropriate responding contractions were obtained. However, an optimized frequency and the necessary time for cells to reach their resting potential had to be furtherly figured out. Additionally, GO‐GelMA hydrogels were synthesized to compare the seeded cells' function in both groups. Higher expression of cardiac markers and better contractility features were noticed on rGO‐GelMA samples, attributed to the enhanced electrical conductivity and physicochemical characteristics of rGO.

**FIGURE 15:**
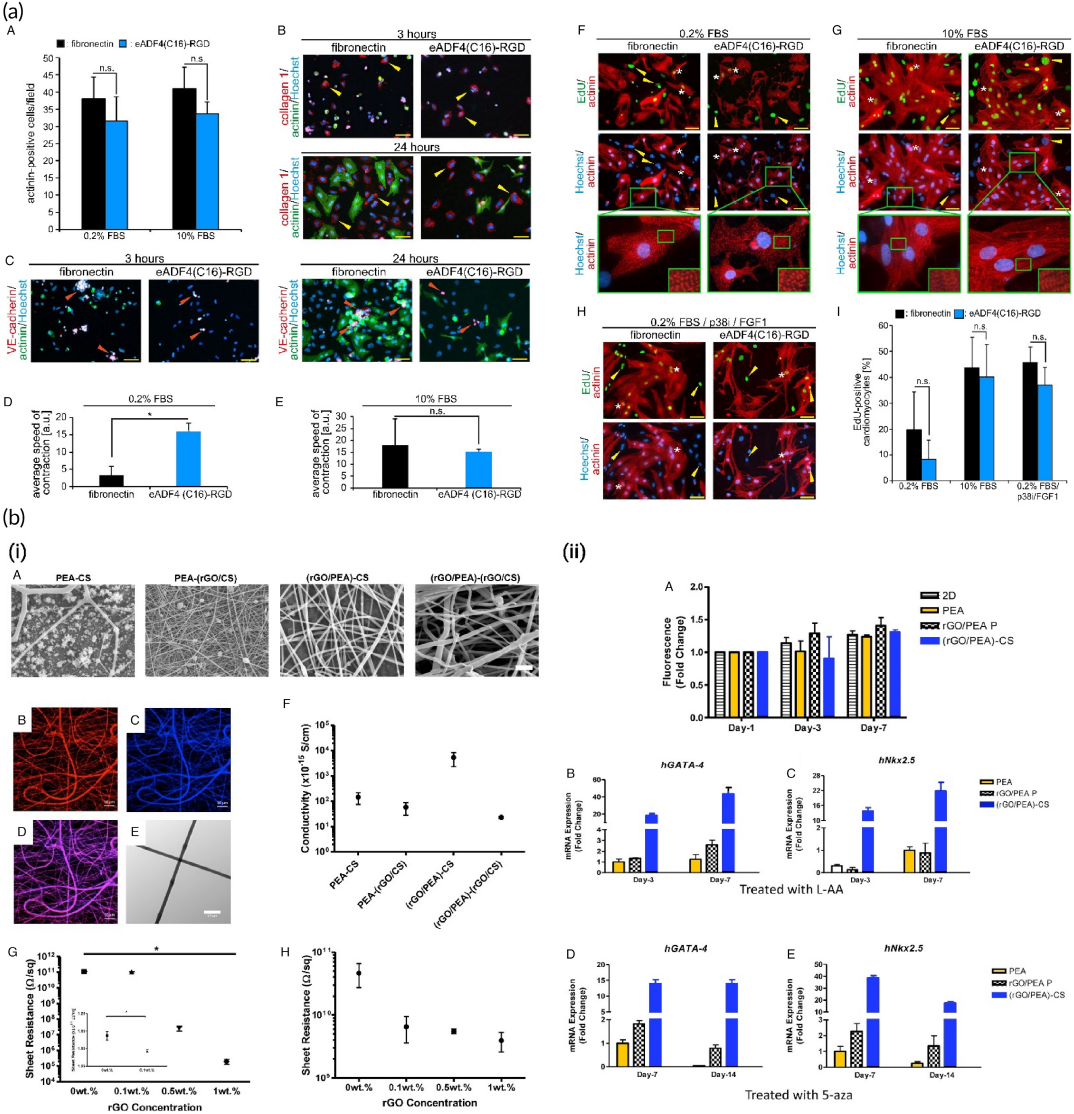
Reduced graphene oxide application in cardiac tissue repair. (a) (A–C) Endothelial, Fibroblast, and CM cells attachments on the eADF4(C16)‐RGD coatings after three‐time points of 3, 24, and 48 h of incubation. (D,E) Quantitative analysis of contraction speed in the presence of 0.2% FBS and 10% FBS, respectively. (F–I) The CM cells were cultured on the eADF4(C16)‐RGD‐based nanostructures for investigating the proliferation and differentiation of the sarcomeres. Reproduced from Reference 318 with permission from Nature. (a) rGO‐poly(ester amide) conductive scaffolds and their potential for cardiac tissue repair. (A) SEM images demonstrating fiber morphology and fiber diameter distribution. (B–D) (rGO/PEA)‐CS (with rGO in the core) where the core is labeled with red DiI (B) and the shell auto‐fluoresces blue (C), and the purple overlap (D) demonstrating the mixing of the core and shell and subsequent lack of clear core‐shell morphology. (E) TEM image indicating non‐homogenous mixing and lack of core‐shell structure. (F) Conductivity of different three‐component coaxial scaffolds. (G,H) The effect of rGO concentration on film resistance of Composite rGO/PEA and rGO/CS films, respectively. (I) Cell proliferation and differentiation on PEA, rGO/PEA P, and (rGO/PEA)‐CS scaffolds. (J–M) iPSC‐derived MSC gene expression. Reproduced from Reference 319 with permission from Elsevier

In another research,^317^ GO‐deposited atop silk fibroin scaffolds, subsequently reduced via ascorbic acid solution, were fabricated for cardiac tissue engineering. As a result of rGO deposition, promoted electrical activity was confirmed by measuring the surface resistance of rGO‐coated samples. Resistivity showed a decreasing manner as the rGO deposited layer thickness increased. Moreover, repeated stretching strains as a simulation of cardiac muscle contraction were applied, and resistance measurements were repeated. Raised values were obtained, plus there was a sharper increase in thicker GO layer coated scaffolds compared to thinner ones. This has been attributed to the lost integrity of the GO layers due to the applied starching cycles and the more fracture potential of thicker layers due to much weaker substrate interface adhesion. Cultured neonatal rat CMs showed enhanced spread and cytoskeleton structure which were likely to relate to several advanced features of rGO/silk scaffolds compared to references, including higher electrical conductivity. Cardiac‐specific markers including α‐actinin, cTnI, and Cx‐43 were further examined on either gene expression or protein organization scale. In comparison to the raw silk, rGO/silk scaffolds exhibited significantly upregulated and promoted expressions. Apart from that, electrical stimulation further increased the expressions. Spontaneous beating characteristics were also assessed. Higher beating rate and intensity were noticed in rGO/silk scaffolds, whether in the presence or absence of electrical stimulation. Interestingly, tissues on bare silk scaffolds showed no difference in beating behavior with or without electrical stimulation, confirming the efficient function of the rGO‐containing deposited layer. Also, the role and interactions between the engineered spider silk protein on the neonatal rat heart cells and the tripeptide‐based nanostructures have been investigated. In this regard, the fibronectin coatings were cultured for 48 h, and the cells were fixed for investigating the CMs' markers including sarcomere alpha‐actinin. The results were interesting and showed that the RGD‐based spider silk proteins on the neonatal rat heart were promising in terms of CMs' attachments. Also, there is no significant difference between the synthesized/prepared nanostructure and the fibronectin coatings in terms of the number of actinin‐positive groups (Figure 15a).^320^

Collagen scaffolds coated with covalently grafted GO flakes, in the form of both partially reduced and nonreduced, were fabricated and compared either in vitro or in vivo as a cardiac patch by Norahan and colleagues.^188^ Both scaffolds were assessed for cytotoxicity, though reduced scaffolds were only evaluated for gene expression and angiogenesis. An average value of 10^−4^ Sm^−1^ was reported for the scaffolds' electrical conductivity in which upregulated expression of TrpT‐2, Cx43, and Actn4 were observed. This was mainly attributed to the facilitated signal propagation due to rGO flakes and improved cell–ECM interactions.

An almost similar study^7^ with much higher concentrations of GO flakes, subsequently reduced, was conducted later through which antibacterial properties were also attained, and electrical properties increasing conductivity values were observed with higher rGO concentrations while all samples showed a conductivity in the range of semiconductors, and the highest conductivity was attributed to the highest reduced GO concentration amount, which was 1.2 ± 0.4 × 10^−4^ Sm^−1^. Results were practically consistent with the previous study.

Thermoresponsive rGO‐incorporated gellan gum (GG) hydrogels were recently investigated by Zargar et al.^226^ as injectable hydrogels aiming for myocardial tissue repair. They showed that the electrical conductivity of anionic GG hydrogels was significantly enhanced as rGO particles' concentrations increased. Cytotoxicity assay also showed no concerns about the prepared hydrogels.

Another study was performed by Stone et al.,^319^ studying conductive fibrous scaffolds made up of poly(ester amide) (PEA) alone or in combination with chitosan (CS), along with rGO particles. PEA or CS solutions and rGO‐containing PEA or CS solutions were used for electrospinning. Conductivity measurements showed (rGO‐PEA)‐CS scaffolds possessed enhanced conductivity, although other groups, including PEA‐(rGO‐CS) and (rGO‐PEA)‐(rGO‐CS) scaffolds, showed decreased conductivities, all compared to control scaffolds of PEA‐CS. This has been likely to be related to the poor dispersion of rGO particles in CS solution. The electrical resistance of rGO‐containing prepared films of both PEA and CS was measured in advance. Consistent results were obtained for rGO‐CS films as well. For cell differentiation assessments, iPSC‐derived MSCs were cultured on (rGO‐PEA)‐CS and rGO‐PEA compared to new PEA scaffolds. Notably, cardiac differentiating factors in order to promote cardiac differentiation were employed. Both rGO‐containing scaffolds strongly supported cardiac differentiation as upregulation of early markers of cardiac differentiation, GATA‐4, and Nkx2‐5 (Figure 15b).

Silk fibroin (SF) containing rGO scaffolds was also created and evaluated by Nazari and colleagues.^321^ A conductivity of 2.01 × 10^−9^ ± 3.6 × 10^−10^ Scm^−1^ was calculated, which was significantly higher compared to the control SF scaffolds (5.99 × 10^−11^ ± 1.2 × 10^−11^ Scm^−1^). TBX18‐transfected hiPSCs were assessed for gene expression following 7 days of culture. RT‐PCR evaluated C‐TNT, α‐MHC, and GATA‐4 expression levels. Upregulation was confirmed for each, compared to control cells. Hence induced cardiac differentiation was achieved as a result of rGO incorporation.

Another study recently performed by Wang et al.^158^ confirmed the great potential of partially reduced graphene oxide for cardiac repair. A 3D foam chip made up of partially reduced GO with CMs seeded supported spontaneous beating of cells within 24 h post cell seeding. Prolonged cultivation time also resulted in more CMs beating in a more synchronized manner. Noteworthy, an average value of 1.12 Scm^−1^ was obtained for the electrical conductivity of the fabricated foam.

### 5.4 Carbon nanotube application in cardiac repair

Compared to graphene, CNTs and CNFs were much earlier introduced to the field of cardiac regeneration. For the first time, purified single‐walled carbon nanotubes were assessed for biocompatibility with cardiac muscle cells by Garibaldi.^322^ H9c2 cells were cultured in a CNT‐containing medium. Cell behavior was characterized and compared to that of untreated cells. Cell growth, survival, viability, and apoptosis were evaluated. An overall view of CNT‐treated cell behavior was obtained, indicating satisfying short‐term biocompatibility while long‐term inconsistency was likely to result in physical rather than chemical interactions.

After the work of Garibaldi, CNTs were extensively employed as fillers for various materials applied in cardiac tissue engineering. Table 4 shows the wide range of their potential applications in polymer fibers or scaffolds, which might in the future revolutionize the CVDs treatment.

**TABLE 4:** The range of potential applications of CNTs in polymer fibers or scaffolds

Conductive substrate	Properties	Conductivity or resistance	Biological effect	Reference
Polydimethylsiloxane/multiwall carbon nanotubes	Microporous and self‐standing	1–4 MΩ	Increase of connexin‐43 gene expression, gap junction areas	323
Polyester–carbon nanotube	Moldable, elastomeric	0.08 ± 0.01 mSm^−1^	Increase the cardiac cell proliferation	96
Polycaprolactone carbon nanotube	3D printed, biodegradable	1.2 × 10^−6^ Scm^−1^	Increase the cardiac cell proliferation	324
CNT‐polyurethane	Interconnected web‐like structures	2.13 × 10^−2^ Scm^−1^	Suitable cytocompatibility for H9c2 cells and human umbilical vein endothelial cells	325
CNTs/aligned poly(glycerol sebacate):gelatin (PG)	Electrospun nanofibers	N/A	Stronger spontaneous and synchronous beating behavior	326
Polyurethane/chitosan/CNT	Aligned electrospun nanofiber, young modulus 4.34 MPa	0.170 kΩ S^−1^	Proper biocompatibility and cell attachment	327
Chitosan‐PVA‐CNT	Elastic modulus: 130 ± 3.605 MPa	3.4 × 10^−6^ Scm^−1^	Cell viability >80%, containing 1% of CNT has optimal properties for cardiac differentiation, the expression of Nkx2.5, Troponin I, and β‐MHC cardiac marker was increased significantly	328

Another study^329^ was performed to determine multi‐walled carbon nanotube's blood compatibility through composition with polyurethane (PU) for cardiovascular surgeries. Platelet activation and red blood cell disruption were observed to be remarkably induced in comparison to pristine PU. Suitability of CNT incorporation for blood‐contacting applications was claimed as a consequence.

As a result of CNT incorporation, electrical conductivity was further considered in a study performed by MacDonald and company,^330^ in which CNT‐embedded collagen composites were prepared to aim for conductivity induction and mechanical benefits. Neural and Cardiac muscle tissue regeneration was intended; however, the electrical conductivity of the constructs was not quantitated. Another study^331^ was later performed, including electrical conductivity measurements. CNT embedded Collagen type I substrates were created targeting electroactivity. More CNT content was observed to result in higher electrical conductivity. On a scale of mS cm^−1^, calculated conductivity varied in the range of 3–7, as the CNT content was leveled up. The obtained electrical properties were suggested to be favorable for the purpose of tissue engineering especially neural and cardiac.

Loads of studies were accordingly performed in cardiac repair, taking advantage of CNT electrical properties. Taking a look at more recently performed studies, Sun et al.^332^ initially reported an investigation on the precise mechanisms involved in the role of CNT incorporation for cardiac regeneration. Single‐walled CNTs at different concentrations were embedded within collagen substrates, and neonatal rat ventricular myocytes were seeded. Pure collagen was used as control, and conductivity measurements confirmed the role of CNTs in improvements of conductivity. An optimal concentration of CNTs for cell viability was obtained through live/dead staining. Samples of 0.1 mg ml^−1^ CNT were chosen for cellular assessments, which display a conductivity value of (1.72 ± 0.31) × 10^−9^ Ω^−1^. Higher cell retention, wider cell spread, thicker actin fibers, and higher adhesion area of cells were observed due to CNT addition to collagen substrate. Cell structures and phenotypes were assessed, and CNT‐col cultured cells were observed to be significantly more maturing. Moreover, marker proteins associated with particular structures forming intercalated discs were examined. Significantly higher levels of the marker proteins including Cx43, N‐cadherin (NC), plakophiilin2 (PKP2), and plakoglobin (PG) were reported at different time intervals up to 14 days past cell cultivation on CNT‐col rather than col substrates. In addition, the ultra‐microstructure of ID assembly was also examined in both experimental groups, and consistent results were noticed. Therefore, the role of CNTs in inducing IDs assembly was concluded. Both groups exhibited spontaneous beating activities; however, more consistency was evidenced in cells within CNT‐col patches. Calcium ion transients and amplitudes are also reportedly promoted, given the presence of CNTs. Besides, activated β1‐integrin signaling is responsible for the enhanced IDs development in the presence of CNTs. Upregulated GATA4 and MEF‐2c were also attributed to CNTs incorporation and suggested to play a role in Cx43 protein expression, thus IDs assembly. As concluded, the electrical conductivity, the particular obtained mechanical strength, and the particular nanotopography features of CNTs are likely to be responsible for such effects.

The same group of researchers^333^ later attempted to examine the potential of these substrates on brown adipose‐derived stem cells' cardiac differentiation. Same single‐walled CNT‐collagen composite scaffolds were fabricated, exhibiting remarkably higher electrical conductivity than pure collagen substrates. Consistent results were indicated overall. They reported an improved cell attachment, proliferation, and cardiogenesis due to CNT incorporation without any induction such as growth factors. Enhanced sarcomeric organization and assembly of gap junctions were observed facilitating matured CMs derived from BASCs. Improved contractile activities were also achieved. Activation of the β1integrin‐dependent TGF‐β1 signaling pathway was evidenced as well as inducing cardiac differentiation within the CNT‐containing substrate. GelMA substrates comprising CNTs were studied as well, yielding consistent results.^334^

They later extended their two‐dimensional works to three‐dimensional constructs to examine whether functional tissues were attainable.^335^ Carboxylic functionalized single‐walled CNTs and collagen hydrogel were synthesized while neonatal rat ventricular myocytes were embedded during the gelation phase of collagen. A conductivity value higher than the native myocardium was calculated for the CNT/col matrix, significantly higher than pure collagen. Consistent and promising results were achieved approving the great potential of the fabricated CNT/col matrix.

Ahadian et al.^336^ also employed GelMA and synthesized GelMA‐aligned CNT gels via the dielectrophoresis approach. Conductivity measurements showed remarkably higher and concentration‐dependent values for GelMA‐aligned CNT samples compared with GelMA‐random CNT and pure GelMA samples. This was due to the presence of CNTs as well as the parallel applied electrical field. Gene and protein expressions were characterized after 4 days of EBs' cultivation and under a continuous 2 days long electrical stimulation. Cardiac genes of Tnnt2, Nkx2‐5, and Actc1, as well as the cardiac protein of Troponin T expressions, were all significantly elevated. This was observed to be even more in the case of electrically stimulated experimental groups. The overall beating activity was also more significant in CNT‐containing groups and was further increased in the presence of the ES.

CNTs directly dispersed in EBs were also studied,^337^ in which a direct relationship between the electrical conductivity and the CNT concentration was observed. As reported, cardiac differentiation and beating activities were considerably induced in EB‐CNTs compared to EBs both in the absence and presence of ES.

More studies using modified unique structures based on CNTs were evaluated. Sheets of super aligned CNTs (SA‐CNTs), for instance, were fabricated by Ren et al.^338^ A highly oriented structure was shown to support anisotropic properties, including anisotropic conductivity, which is reported to be 10 times higher along with the structure across the transverse direction. Randomly dispersed CNT (RD‐CNTs) sheets and cover glasses were also investigated as control groups, and neonatal rat CMs were cultured, showing the great potential for cardiac repair.

Probing more anisotropic structures mimicking the native microstructure of the myocardium, 3D layered structures of GelMA hydrogels encapsulating a composition of PCL and silk fibroin containing CNTs were fabricated by Wu and colleagues.^113^ An orthogonal direction was imposed between the layers observed to support the maturation and alignment of CMs.

Tondnevis et al.^339^ conducted a study on the potential of a polyurethane electrospun scaffold to support either endothelial and myocardial myoblast cells for cardiovascular tissue engineering. Gelatin and single‐walled CNTs were additionally utilized to improve the biological and electrical properties, respectively. The scaffold's innovative composition and nanofibrous structure and a mean conductivity of 1.3 × 10^−2^ ± 5 × 10^−3^ S cm^−1^ showed excellent potential as cell proliferation and adhesion were significantly enhanced.

In another study,^328^ CNT‐containing electrospun scaffolds were synthesized based on chitosan and polyvinyl alcohol, and the calculated value for electrical conductivity was 3.4 × 10^−6^ S cm^−1^.

A 3D printed conductive cardiac patch with an electrical conductivity of 4.3 × 10^−1^ S cm^−1^ was also made.^340^ However, increased values of conductivity were noticed as a function of the wetting state. The obtained values were higher than the required electrical properties designated for the native myocardium. Thus, a perfect contribution of the patch and the scar region in order to compensate for scar‐related arrhythmias. In vivo evaluations were conducted to corroborate their hypothesis.

**FIGURE 16:**
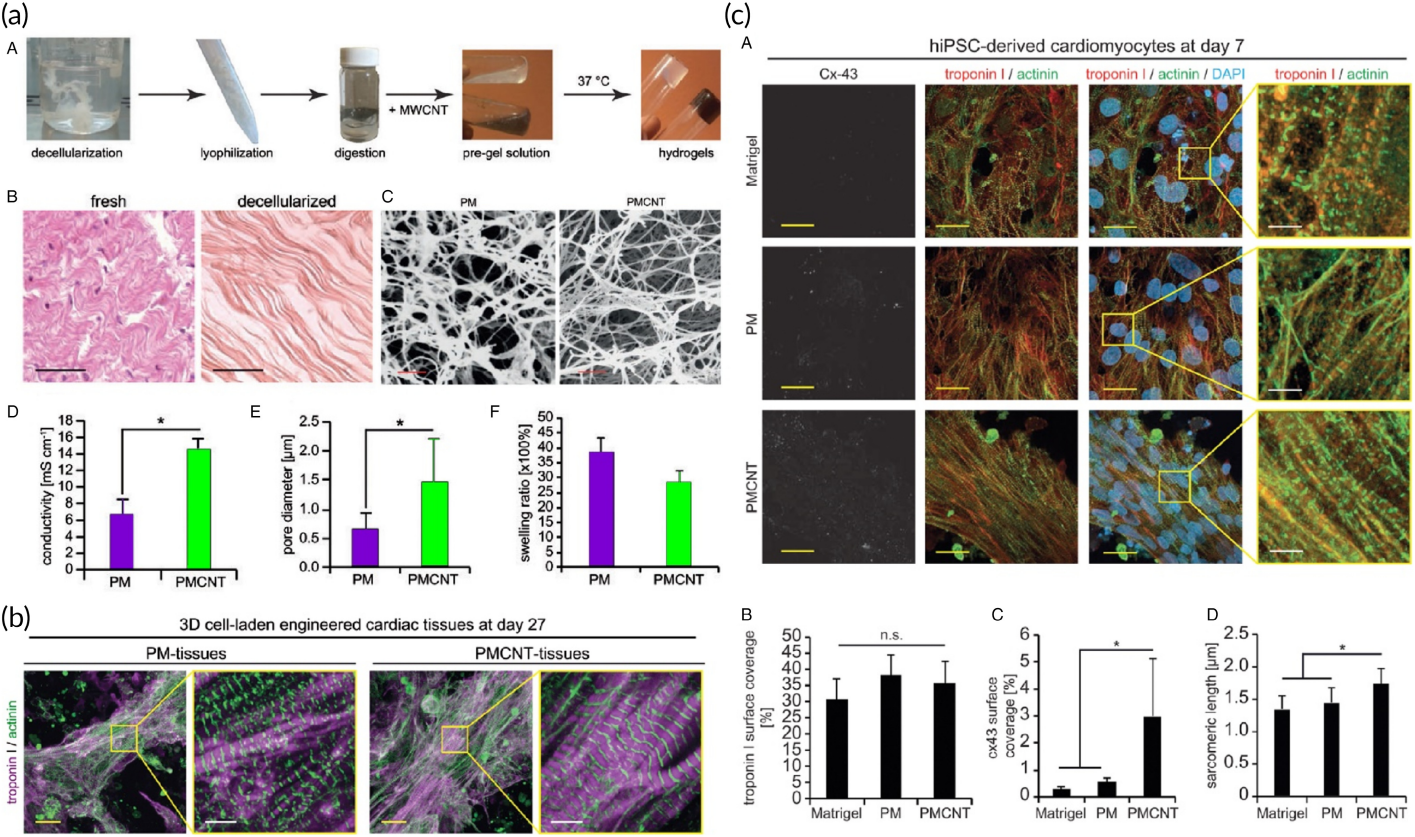
Carbon nanotube adorned hydrogel for cardiac tissue engineering. (a) (A) Illustration of different steps in preparation of pericardial matrix (PM)‐ and PMCNT‐gels. (B) hematoxylin–eosin staining histological images of the fresh and decellularized pericardium. (C) SEM images of PM‐ and PMCNT‐gels. (D–F) Quantitative analyses of electrical conductivity, pore diameter, and swelling ratio, respectively. (b) confocal images of cell‐laden tissue construct stained for the CM‐specific markers troponin I and sarcomeric‐α‐actinin. (c) Fabricated hydrogels intrinsically increase intercellular electrical coupling of hiPSC‐derived CMs. Reproduced from Reference 341 with permission from The Royal Society of Chemistry

An innovative study carried out by Roshanbinfar et al.^341^ utilized functionalized multi‐walled CNTs. The pericardial matrix of sheep was decellularized and enzymatically digested before CNT addition. Significant enhancement was measured for the electrical conductivity of the prepared gel, reported to be 1.42 × 10^−2^ ± 1.2 × 10^−3^ S cm^−1^. Claiming the fabricated hydrogel to be a suitable substrate for hiPSC‐derived CMs, autonomous synchronized contractions are reported while arrhythmic beats were apparent in the cases of CNTs absence and the Matrigel groups. Also included are more efficient contractions, improved calcium handling properties, elevated Cx43 expression, and increased sarcomeric length (Figure 16).

Lee et al. later performed a comparison study^342^ on CNT, GO, and rGO‐containing scaffolds based on GelMA. The electrical resistivity measurements indicated low resistance for rGO (∼1 kΩ/sq) and CNT particles (∼100 kΩ/sq), while GO particles showed relatively no electrical conductivity. More cell spreading area, more elongated cell shapes, better cell retention, higher levels of vinculin, Cx43, and Troponin I expression, longer sarcomeric α‐actin, more ventricular‐like phenotypes, lower excitation threshold, along with proper beating behaviors of cultured neonatal rat CMs are reported. The obtained results are attributed to the promoted electrical conductivity and favorable mechanical, structural, physical, and chemical properties of CNT‐containing GelMA scaffolds. In a very recent study, a silk fibroin‐based CNT‐containing scaffold was also fabricated by Zhoa et al., a fully aqueous approach was employed, guided cardiac regeneration was intended, and promising results as a function of CNT incorporation and enhanced electrical conductivity were demonstrated. Notably, a growing amount of conductivity was obtained as a function of CNT concentration, ranging from 2.5 × 10^−7^ to 1.5 × 10^−6^ Scm^−1^. These results showed the capability of CNT‐based scaffolds in improving the conductivity of the potential cardiac tissue engineering.^343^, ^344^

Wu et al. fabricated a 3D patch to mimic the structure of the native tissue. Mimicking the anisotropic cardiac structure and guiding 3D cellular orientation is critical in designing scaffolds for cardiac tissue regeneration. Significant advances have been achieved to control cellular alignment and elongation, but these approaches remain an ongoing challenge for engineering 3D cardiac anisotropy. They fabricated a 3D hybrid scaffold based on an aligned conductive nanofiber yarns network (NFYs‐NET, composition: polycaprolactone, silk fibroin, and carbon nanotubes) within a hydrogel shell for mimicking the native cardiac tissue structure and further demonstrate their great potential for engineering 3D cardiac anisotropy for cardiac tissue engineering. The NFYs‐NET structures are shown to control cellular orientation and enhance CMs' maturation. 3D hybrid scaffolds were then fabricated by encapsulating NFYs‐NET layers within hydrogel shells, and these 3D scaffolds performed the ability to promote aligned and elongated CMs maturation on each layer and individually control cellular orientation on different layers in a 3D environment. Furthermore, the endothelialized myocardium was constructed using this hybrid strategy via the coculture of CMs on the NFYs‐NET layer and endothelial cells within the hydrogel shell. Therefore, these 3D hybrid scaffolds, containing NFYs‐NET layer inducing cellular orientation, maturation, anisotropy, and hydrogel shell providing a suitable 3D environment for endothelialization, have great potential in engineering 3D cardiac anisotropy.^113^

### 5.5 Carbon nanofiber application in cardiac repair

Carbon nanofibers combined with different biomaterials were also earlier used for various tissue regeneration studies.^345^, ^346^ However, its entry into the cardiac area was delayed until Stout et al.^194^, ^347^ made the first use of its conductivity and cytocompatibility. Myocardial tissue repair induction potency of Poly(lactic‐co‐glycolic acid) (PLGA)‐CNF composites were evaluated. Evaluations approved the ascending behavior of electrical conductivity as a result of increased CNF weight ratios within composites. Human CMs and rat neuroblastoma cells were cultured for in vitro cell culture assays. Results showed that cells density, as well as their proliferation rate, were both remarkably increased within composites referenced to pure PLGA. The promising results were likely to be attributed to the fabricated substrates' specific topography, roughness, and favorable electrical properties.

Later, they conducted a modified version of the previous study, utilizing a continuous electrical stimulation for 1, 3, and 5 days.^348^ Cytocompatibility and viability assays were conducted on cultured human CMs, and the same overall trends were recorded. Interestingly, slightly promoted behaviors in all samples were observed as a consequence of electrical stimulations.

Further studies were conducted aiming to investigate the function and the mechanism of observations more precisely. Cardiac differentiation markers including troponin T, connexin 43, and α‐SMA were seen to be highly expressed on prepared CNF‐containing samples.^157^ However, a descending manner of expression was observed in concentrations above some specific values.

Moreover, various cardiovascular cell types were also assessed for growth characteristics cultures on PLGA‐CNF substrates.^349^ Previously obtained results supported CMs through hindering effects on fibroblast, and endothelial cell growth was observed for both non or electrically stimulated groups. As indicated, this could have to do with the potential to impede the growth of fibrosis and noncontractile cells while favorably supporting CMs growth. However, the mechanism was not precisely figured out.

In another study,^350^ the unique anisotropic structure of the native myocardium was regarded. Accordingly, aligned CNF‐PLGA composites were fabricated. In this regard, a voltage was applied to the CNF containing the solution of PLGA before setting, thus achieving a proper orientation of nanofibers. Vertical and horizontal conductivity values of 1 × 10^−3^ and 2.5 × 10^−5^ Scm^−1^ were calculated, while randomly dispersed CNF samples showed the same 7.5 × 10^−4^ Scm^−1^ in both directions. Better adhesion and a high rate of cell proliferation were achieved on anisotropic substrates, presumably due to the specific established electrical and mechanical properties, which further improve cell‐to‐cell communications.

The preliminary studies aforementioned typically opened new horizons for CNF applications in the field of cardiac repair. So far, CNFs have been studied in combination with various materials including pHEMA (poly(2‐hydroxyethyl methacrylate)),^351^ chitosan,^60^ gelatin,^352^ and collagen^353^ representing different conductivity values of 1.8 × 10^−3^ Ω^−1^,^351^ 2.5 × 10^−3^ ± 9 × 10^−4^ S cm^−1^,^60^ and 8.39 × 10^−2^ ± 1.2 × 10^−7^ S cm^−1^.^352^ Taken together, presumed potentials and previously suggested mechanisms were supported.

Martins et al.^60^ reported cardiac markers expression profiles through real‐time qPCR, indicating a significant increase in Tnnc1, Cx43, And, Myh6, Myh7, GATA4, and Atpa2a gene expressions in CNF‐chitosan scaffolds compared to the new chitosan control group.

In addition to gene expression evaluations, Mehrabi et al.^352^ performed subcutaneous implantation of fabricated patches aiming at angiogenic potency quantifications. Histology and immunohistochemistry observations showed more remarkable cell migration, and more capillaries were detected.

In a very recent study,^353^ MI‐induced rats were treated with fabricated collagen‐based scaffolds. Less immunogenicity, a smaller number of dead cells and more live cells, and induced angiogenesis due to CNFs incorporation have been reported.

## 6 CONCLUSION AND FUTURE PERSPECTIVES

CVDs are the leading cause of death worldwide. Since decisive treatments for CVDs can change the statistics and human life expectancy, attempts at cardiac repair are of tremendous importance. Myocardial repair following MI is a topic of interest as a result. In this approach, regenerative medicine is now looked upon as a grand promise for overcoming the main limitations of the intrinsic incapability of the human body for cardiac repair. However, significant hurdles remain, which await further investigations.

Different demands have to be met, aiming to create a functional construct. Appropriate cell selection, substrate material, and other molecular components including mechanical, and electrical properties are the influential parameters, all of which have to be taken into account. Electrical properties were mainly considered in this literature. Conductive substrates, which can integrate into the native myocardium are great potential for which signal propagation is possible. Carbon‐based materials are likely to be promising candidates among different conductive materials as they can simultaneously offer multiple benefits, including electrical conductivity. A review of recent studies utilizing different conductive polymers was performed to give a broad view of the mission of conductivity in cardiac tissue engineering. Then, carbon‐based materials and biomaterials were reviewed, considering their conductivity impacts on myocardial repair. Such a broad view may make those working in the field aware of the state of cardiac repair; moreover, provide them with a comparative view of the use of each polymer and carbon‐based nanoparticle in their families. Increased expression of several cardiac‐specific markers, induced cardiac differentiation of stem cells, promoted contractile behavior and beating activities of CMs, and enhanced cell proliferation and cell adhesion were obtained in almost all in vitro studies. Improved cardiac function and decreased fibrotic areas have also been observed in vivo.

Taken together, carbon‐based materials seem to be the perfect choice to provide the essential electroactivity in cardiac tissue scaffolds. However, the potential cytotoxicity, genotoxicity, and carcinogenicity of these nano‐sized materials in the presence of cells within the myocardium environment and even after degradation is still a topic of much debate and discussion. More thorough investigations, in vivo studies, and clinical trials are accordingly required to better optimize and accomplish the most proper and safe combination of materials for cardiac regenerative therapies in this respect.

## AUTHOR CONTRIBUTIONS

**Negin Jalilinejad:** Writing – original draft (equal). **Mohammad Rabiee:** Writing ‐ editing the final version (equal). **Nafiseh Baheiraei:** Writing – original draft (equal). **Reza Salarian:** Writing ‐ editing the final version (equal). **Ramin Ghahremanzadeh:** Writing – original draft (equal). **Navid Rabiee:** Writing ‐ original draft (equal). **Omid Akhavan:** Writing – original draft (equal). **Aleksander Hejna:** Writing – original draft (equal). **Mohammad Reza Saeb:** Writing ‐ editing the final version (equal). **Ali Zarrabi:** Writing ‐ editing the final version (equal), visualizations. **Esmaeel Sharifi:** Writing ‐ editing the final version (equal), visualizations. **Satar Yousefiasl:** Writing ‐ editing the final version (equal), visualizations. **Ehsan Nazarzadeh Zare:** Writing ‐ editing the final version (equal).

## CONFLICT OF INTEREST

The authors declare no conflict of interest.

### PEER REVIEW

The peer review history for this article is available at https://publons.com/publon/10.1002/btm2.10347.

## Data Availability

Data sharing is not applicable to this article as no new data were created or analyzed in this study.
